# Hepatic deficiency of selenoprotein S exacerbates hepatic steatosis and insulin resistance

**DOI:** 10.1038/s41419-022-04716-w

**Published:** 2022-03-28

**Authors:** Lu Qiao, Lili Men, Shanshan Yu, Junjie Yao, Yu Li, Mingming Wang, Ying Yu, Ning Wang, Liyuan Ran, Yingjie Wu, Jianling Du

**Affiliations:** 1 Department of Endocrinology, the First Affiliated Hospital of Dalian Medical University, Dalian, China; 2 Dalian Key Laboratory of Prevention and Treatment of Metabolic Diseases and the Vascular Complications, Dalian, China; 3 Institute for Genome Engineered Animal Models of Human Diseases, Dalian Medical University, Dalian, China; 4 National Center of Genetically Engineered Animal Models for International Research, Dalian Medical University, Dalian, China; 5 Department of Molecular Pathobiology, New York University College of Dentistry, New York, NY USA

**Keywords:** Metabolic syndrome, Obesity

## Abstract

Nonalcoholic fatty liver disease (NAFLD) is closely associated with insulin resistance (IR) and type 2 diabetes mellitus (T2DM), which are all complex metabolic disorders. Selenoprotein S (SelS) is an endoplasmic reticulum (ER) resident selenoprotein involved in regulating ER stress and has been found to participate in the occurrence and development of IR and T2DM. However, the potential role and mechanism of SelS in NAFLD remains unclear. Here, we analyzed SelS expression in the liver of high-fat diet (HFD)-fed mice and obese T2DM model (*db/db*) mice and generated hepatocyte-specific SelS knockout (*SelS*^*H-KO*^) mice using the Cre-loxP system. We showed that hepatic SelS expression levels were significantly downregulated in HFD-fed mice and *db/db* mice. Hepatic SelS deficiency markedly increased ER stress markers in the liver and caused hepatic steatosis via increased fatty acid uptake and reduced fatty acid oxidation. Impaired insulin signaling was detected in the liver of *SelS*^*H-KO*^ mice with decreased phosphorylation levels of insulin receptor substrate 1 (IRS1) and protein kinase B (PKB/Akt), which ultimately led to disturbed glucose homeostasis. Meanwhile, our results showed hepatic protein kinase Cɛ (PKCɛ) activation participated in the negative regulation of insulin signaling in *SelS*^*H-KO*^ mice. Moreover, the inhibitory effect of SelS on hepatic steatosis and IR was confirmed by SelS overexpression in primary hepatocytes in vitro. Thus, we conclude that hepatic SelS plays a key role in regulating hepatic lipid accumulation and insulin action, suggesting that SelS may be a potential intervention target for the prevention and treatment of NAFLD and T2DM.

## Introduction

Nonalcoholic fatty liver disease (NAFLD) affects up to 25% of the population worldwide and poses tremendous threats to health and quality of life [1]. It is a chronic liver disease linked to insulin resistance (IR) and genetic susceptibility. The hallmark of NAFLD is hepatic steatosis, and the disease develops by excessive accumulation of triglyceride (TG) in hepatocytes attributed to hepatic TG synthesis occurring more rapidly than TG disposal [2]. NAFLD often coincides with hepatic IR and increases the risk of fasting hyperglycemia and type 2 diabetes mellitus (T2DM) [3–5]. In the liver, insulin action via activating a signaling pathway including insulin receptor substrate (IRS) and protein kinase B (PKB/Akt) is crucial for the regulation of glucose metabolism [6]. Therefore, attenuating hepatic lipid accumulation and IR can effectively improve glucose, and lipid metabolic disorders.

Mounting evidence has shown that selenoprotein S (SelS) plays roles in obesity, IR, glucose and lipid metabolism [7–13]. SelS has extensive histological distributions coupled with tissue-specific biological functions. Overexpression of SelS protects Min6 pancreatic β-cells and vascular endothelial cells from oxidative stress [10, 14], while SelS expression in the liver, adipose tissue and skeletal muscle promotes the occurrence and development of IR and diabetes [9, 13, 15]. Our group has shown that the expression of SelS is increased in the white adipose tissue (WAT) of obese subjects and high-fat diet (HFD)-fed mice, while knockdown of SelS induces 3T3-L1 preadipocyte differentiation defect, indicating that SelS is involved in the pathogenesis of obesity [11]. The above studies demonstrate that SelS is a key regulator in metabolic syndrome.

The disruption of endoplasmic reticulum (ER) homeostasis known as ER stress is essential for the initiation and progression of NAFLD and IR [16–20]. In response to unfolded or misfolded ER proteins, an adaptive unfolded protein response (UPR) is triggered via glucose-regulated protein 78 (GRP78) dissociating from three ER stress sensors, including inositol-requiring enzyme 1α (IRE1α), protein kinase RNA-like ER kinase (PERK) and activating transcription factor 6 (ATF6), thereby activating all signaling cascades to restore ER homeostasis. The UPR reduces ER protein load by enhancing ER-associated protein degradation (ERAD). SelS forms a multiprotein complex with p97 ATPase, Derlin-1, E3 ubiquitin ligase and selenoprotein K to participate in ERAD, mediating the retro-translocation of misfolded or unfolded proteins from the ER lumen to the cytosol for degradation [21–23]. It has been reported that SelS expression is upregulated under the stimulation of ER stress inducer [10, 24–26]. SelS protects macrophages, vascular smooth muscle cells, and astrocytes from apoptosis caused by ER stress [26–28], suggesting that SelS possesses the potential to attenuate the damage in response to ER stress. Given the role of ER stress in NAFLD and IR, the effect and mechanism of SelS in hepatic steatosis and IR remains to be confirmed.

The present study showed the effect of obesity and diabetes on SelS expression by analyzing hepatic SelS expression in HFD-fed mice and *db/db* mice. Hepatocyte-specific SelS knockout (*SelS*^*H-KO*^) mice were generated to investigate the effect and underlying molecular mechanism of SelS on hepatic steatosis and IR. Our findings may provide a novel prevention and intervention strategy for NAFLD and T2DM.

## Materials and methods

### Mouse husbandry and generation of *SelS*^*H-KO*^ mice

All mice were housed in the pathogen-free Animal Center of Dalian Medical University (Dalian, China) under a controlled temperature (23 °C) and 12 h light/dark cycle with *ad libitum* access to water and food. *C57BL/6* wild-type male mice were from the SPF Animal Center of Dalian Medical University. *db/db* and *db/m* male mice were purchased from Laboratory Animal Co., Ltd. (Changzhou, China). Homozygous floxed SelS (*SelS*^*F/F*^) mice were commercially generated by Biocytogen Co., Ltd (Beijing, China) on a *C57BL/6* background. Transgenic *Albumin-Cre* (*Alb-Cre*) mice (B6.FVB(129)-Tg(Alb1-cre)1Dlr/J) were gifted by the Institute for Genome Engineered Animal Models of Human Diseases, Dalian Medical University. Heterozygous mice were produced by crossing *SelS*^*F/F*^ mice with *Alb-Cre* mice. *SelS*^*H-KO*^ mice were generated by crossing heterozygous mice. Only male mice were used in the present experiments. Genomic DNA was extracted from the tail for genotyping (Fig. S1A), and DNA was extracted from various tissues for PCR analysis to confirm the specificity of SelS deletion in the liver (Fig. S1B). Primers are listed in Table S1. After adapting to the environment for 4 weeks, both *SelS*^*F/F*^ and *SelS*^*H-KO*^ mice were randomized into two groups: one group was kept on regular chow (D12450J, 10% kcal from fat, 20% kcal from protein, 70% kcal from carbohydrate, Research Diets), and the other group was transferred to a HFD (D12492, 60% kcal from fat, 20% kcal from protein, 20% kcal from carbohydrate, Research Diets) for 20 weeks. Body weight was monitored once a week during this period. Blood was collected from the eyeballs of 24-week-old mice. After dissection, tissues were weighed and then fixed with 10% formalin solution or snap-frozen in liquid nitrogen before stored at −80 °C. All animal experiments were approved by the Animal Experimental Ethics Committee of Dalian Medical University.

### Glucose tolerance test (GTT) and insulin tolerance test (ITT)

Mice were fasted for 12 h overnight, and then the GTT was carried out by intraperitoneal injection of glucose (2 g/kg body weight). Blood glucose levels were measured at 0, 15, 30, 60, and 120 min with glucometers (Roche, Mannheim, Germany). After fasting for 5 h, mice were injected intraperitoneally with insulin (0.75 IU/kg body weight) for the ITT. Blood glucose levels were measured with glucometers (Roche) at 0, 15, 30, and 60 min.

### Serum analysis

Serum was separated from whole blood by centrifugation at 3000 rpm for 15 min at 4 °C. Serum samples were used to analyze serum lipid levels, including TG, total cholesterol (TC), high-density lipoprotein cholesterol (HDL-C), low-density lipoprotein cholesterol (LDL-C) and free fatty acid (FFA), with corresponding colorimetric kits (Jiancheng, Nanjing, China). The serum levels of alanine aminotransferase (ALT) and aspartate aminotransferase (AST) were measured with colorimetric kits (Jiancheng). Quantitative measurements of serum fibroblast growth factor 21 (FGF21) (Boster, Wuhan, China), adiponectin (Cloud-Clone Corp, Wuhan, China) and insulin (Cloud-Clone Corp) were performed using mouse enzyme-linked immunosorbent assay (ELISA) kits, respectively. All procedures were performed in accordance with the instructions of the manufacturer.

### Hematoxylin-eosin (H&E) staining and Oil Red O staining

Liver and adipose tissues were fixed with 10% formalin solution for 24 h. Paraffin-embedded tissues were sectioned at a thickness of 5 μm and stained with H&E. Optimal cutting temperature (OCT)-embedded tissues were sectioned at a thickness of 8 μm, rinsed with 60% isopropanol and then stained with Oil Red O (Solarbio, Beijing, China). Primary hepatocytes were fixed with 10% formalin solution for 30 min, and then stained with Oil Red O (Solarbio).

### Measurement of hepatic lipid and glycogen content

Lipids in the liver were extracted as previously described by Folch et al. [29] in chloroform: methanol (1:2, vol/vol). Liver diacylglycerol (DAG) content was measured using the Mouse DAG ELISA kit (Lengton, Shanghai, China). TG content and glycogen content in the liver were measured with corresponding colorimetric kits (Jiancheng). All procedures were performed following the manufacturer’s protocols.

### Primary hepatocyte isolation, culture and study design

Primary hepatocytes were isolated from regular chow (RC)-fed 8-week-old male mice. Before hepatocyte isolation, 10 cm dishes were coated with 0.1% gelatin (Sigma, Missouri, USA) for 4 h at room temperature. Mice were anesthetized, and the thoracic and abdominal cavities were exposed. The inferior vena cava was separated, and a needle was inserted. The portal vein was cut, and D-Hanks solution was perfused until the liquid became clear, followed by perfusion with Hanks solution containing 0.025% type IV collagenase (Sigma) for 8-10 min. The gall bladder was removed, and the liver was transferred into a 10 cm dish containing cold Dulbecco’s modified Eagle’s medium (DMEM). The liver was gently dissected, and cells were released. The cell suspension was filtered by a cell strainer (40 μm, Falcon, USA), washed twice in cold phosphate buffer solution and centrifuged at 750 rpm for 3 min at 4 °C to obtain the primary hepatocyte pellets. The primary hepatocytes were cultured in DMEM containing 10% fetal bovine serum, 100 U/ml penicillin, and 100 μg/ml streptomycin in a humidified incubator with 5% CO_2_ at 37 °C.

To investigate the effect of palmitic acid (PA) stimulation on SelS expression, PA powder (Sigma) was dissolved in 400 mM NaOH, and mixed with 10% bovine serum albumin (Sigma), then diluted into DMEM to make a 0.5 mM PA solution. The primary hepatocytes were stimulated with 0.5 mM PA for 24 h, and medium with same concentrations of vehicles served as control. Then, the protein expression levels of SelS were measured.

To investigate the effects of SelS on hepatosteatosis and insulin signaling in vitro, the primary hepatocytes were treated with 0.5 mM PA for 24 h after infected with corresponding adenoviruses for 24 h. To identify the role of protein kinase Cɛ (PKCɛ) in insulin signaling, the primary hepatocytes were preincubated with PKCɛ inhibitor (ε-V1-2, 1 μM, MedChemExpress, New Jersey, USA) or PKCɛ activator (DCP-LA, 100 nM, MedChemExpress) for 1 h after adenovirus infection, and then the hepatocytes were treated with 0.5 mM PA for 24 h. Changes in the phosphorylation levels of IRS1 and Akt in cells were measured.

### Infection of primary hepatocytes with recombinant adenovirus

Adenovirus vectors encoding green fluorescent protein, SelS (overexpression (OE)-CON, SelS-OE), and containing scramble short-hairpin RNA (shRNA) and SelS shRNA (knockdown (KD)-CON, SelS-KD) were purchased from Gene Pharm, Suzhou, China. The sequences were as follows: SelS-KD1 (5′-GGTCCTGGACCTTCTACTTCA-3′); SelS-KD2 (5′-GGACCAAGCCGAGACTGTTCT-3′); KD-CON (5′-TTCTCCGAACGTGTCACGT-3′). Adenovirus infection was performed according to the manufacturer’s instructions. After 24 h post-infection, cells were harvested to test infection efficiency.

### 3T3-L1 preadipocyte culture, differentiation, and study design

3T3-L1 preadipocytes were cultured in DMEM supplemented with 10% HI bovine serum (FCS). Differentiation was initiated as described [11, 12] until more than 80% of cells became mature adipocytes. Then the cells were stimulated with 0.01–200 nM recombinant mouse FGF21 (Abbkine, Wuhan, China) for 24 h. To further identified the effect of FGF21 on adipocytes, the mature adipocytes were preincubated with FGF receptor-1 (FGFR1) inhibitor (PD166866, 10 μM, MedChemExpress) for 1 h before stimulation with 0, 1, 100 nM recombinant mouse FGF21 for 24 h. Both SelS and adiponectin protein levels were measured.

### Western blotting

Tissues and cells were treated with RIPA buffer (Solarbio) to extract proteins. Equal amounts of total proteins were separated by sodium dodecyl sulfate (SDS)‐polyacrylamide gel electrophoresis (PAGE), transferred to nitrocellulose membranes, and incubated with the indicated primary antibodies overnight at 4 °C. The details of the primary antibodies are listed in Table S2. The membranes were incubated with secondary antibodies for 2 h at room temperature and immersed in enhanced chemiluminescence solution (Abbkine). Tubulin or GAPDH was used as an internal reference. The western blotting was finally imaged with Bio-Rad’s imaging system (Bio-Rad, California, USA). Hepatic cytosolic and membrane proteins were prepared according to the method of Kumashiro et al. [30] and used for the detection of PKCɛ translocation. Tubulin or GAPDH and Na^+^/K^+^-ATPase were used as internal references, respectively.

### Real-time quantitative PCR

Total RNA was extracted using the RNAiso Plus kit (Takara, Dalian, China). Reverse transcription reactions were performed using the Prime Script^TM^ RT reagent kit (Takara). Real-time quantitative PCR was carried out with the TransStart Tip Green qPCR SuperMix kit (TransGen, Beijing, China). Relative mRNA expression was calculated using the 2^−ΔΔCt^ method, with *Gapdh* as an endogenous control. The primers used for real-time quantitative PCR are listed in Table S3.

### Statistical analysis

All data are presented as the mean ± standard error of the mean (SEM) and were analyzed using SPSS 23.0 statistical software. Statistical analysis was performed with Student’s *t* test. *P < 0.05 was considered to be statistically significant.

## Results

### Obesity and diabetes downregulate hepatic SelS expression

**Fig. 1:**
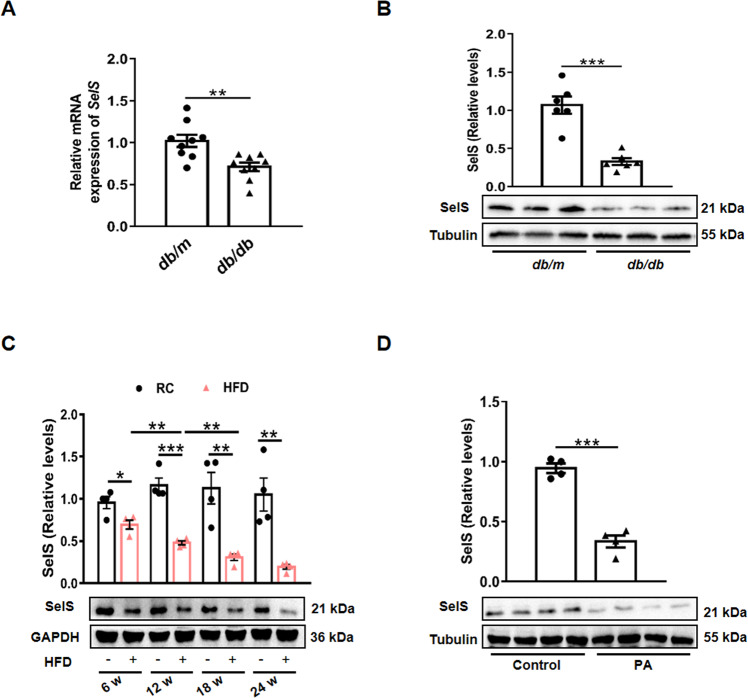
Regulation of SelS expression in the mouse liver and primary hepatocytes. **A** *SelS* mRNA expression in the liver of *db/m* and *db/db* mice (*n* = 9 per group). **B** Representative Western blotting images from 3 mice out of 6 for each group and quantified protein levels of SelS in the liver of *db/m* and *db/db* mice (*n* = 6 per group). **C** Representative Western blotting images from one mouse out of 4 for each group and quantified protein levels of SelS in the liver of wild-type mice fed with RC or HFD for 6 w, 12 w, 18 w, or 24 w (*n* = 4 per group). **D** Western blotting and quantified protein levels of SelS in the primary hepatocytes isolated from wild-type mice treated with 0.5 mM PA for 24 h (*n* = 4 per group). All data are presented as the mean ± SEM. *P < 0.05, **P < 0.01, ***P < 0.001. SelS, selenoprotein S; RC, regular chow; HFD, high-fat diet; PA, palmitic acid.

To determine whether obesity and diabetes affect hepatic SelS expression in mice, SelS expression levels were measured in the liver of obese T2DM model (*db/db*) mice and HFD-fed mice. As shown in Fig. 1A and 1B, SelS mRNA expression and protein levels were lower in the liver of *db/db* mice than in *db/m* mice (*P* < 0.01). Wild-type mice fed with a HFD for 6 w, 12 w, 18 w, or 24 w showed a gradual decrease of hepatic SelS expression in the progression of obesity (*P* < 0.05; Fig. 1C). We further confirmed SelS expression levels were decreased in 0.5 mM PA-treated primary hepatocytes (*P* < 0.001; Fig. 1D). Taken together, these data indicate that impaired metabolic states regulates hepatic SelS expression.

### Hepatic-specific deletion of SelS causes obesity, hepatic steatosis, and dyslipidemia

**Fig. 2:**
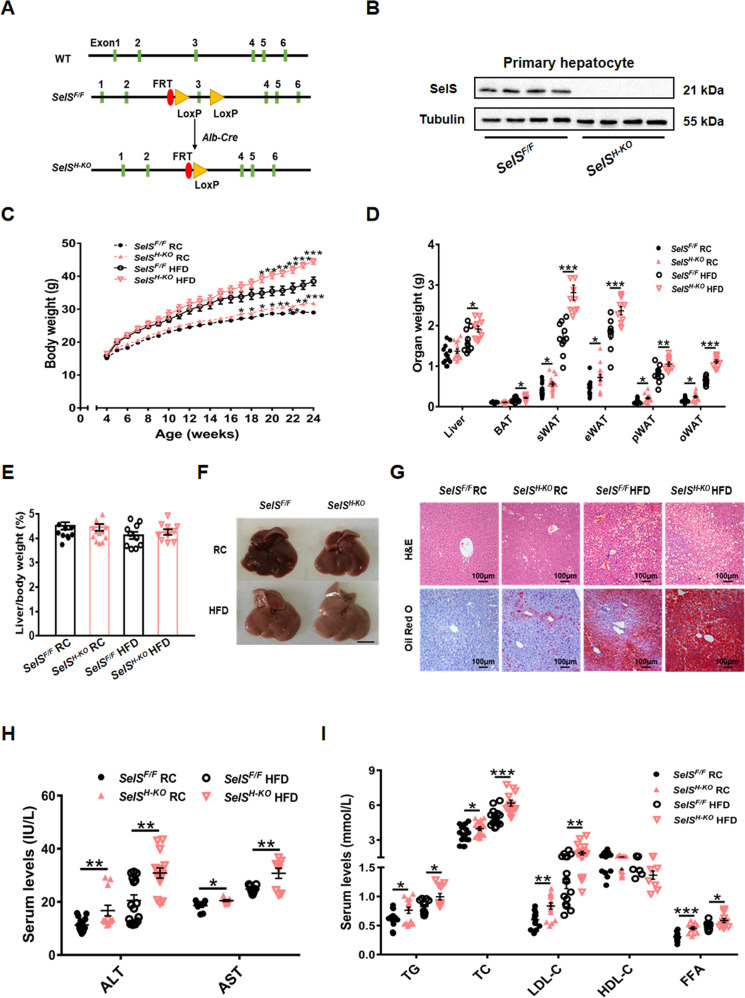
Hepatic-specific deletion of SelS causes obesity, hepatic steatosis and dyslipidemia. **A** Schematic diagram of *SelS*^*H-KO*^ mice construction. **B** Western blotting analysis of SelS protein levels in isolated primary hepatocytes from *SelS*^*H-KO*^ and *SelS*^*F/F*^ mice (*n* = 4 per group). **C** The growth curve of *SelS*^*H-KO*^ and *SelS*^*F/F*^ mice during 20 weeks of RC or HFD feeding (*n* = 10–12 per group). **D** The weight of the liver and each part of the fat pad from 24-week-old *SelS*^*H-KO*^ and *SelS*^*F/F*^ mice fed with RC or HFD for 20 weeks (*n* = 10–15 per group). **E** Liver weight/body weight ratio of the indicated groups (*n* = 10–15 per group). **F** Representative images of liver isolated from mice of the indicated groups. Scale bar, 1 cm. **G** H&E and Oil Red O staining of livers from the indicated groups. Scale bar, 100 μm. **H** Serum ALT and AST levels in the indicated groups (*n* = 9–15 per group). **I** Serum TG, TC, LDL-C, HDL-C and FFA levels in the indicated groups (*n* = 9–18 per group). All data are presented as the mean ± SEM. *P < 0.05, **P < 0.01, ***P < 0.001. RC, regular chow; HFD, high-fat diet; *SelS*^*H-KO*^, hepatocyte-specific SelS knockout mice; *SelS*^*F/F*^, floxed SelS mice; BAT, brown adipose tissue; sWAT, subcutaneous white adipose tissue; eWAT, epididymal white adipose tissue; pWAT, perirenal white adipose tissue; oWAT, omental white adipose tissue; H&E, hematoxylin and eosin; ALT, alanine aminotransferase; AST, aspartate aminotransferase; TG, triglyceride; TC, total cholesterol; LDL-C, low-density lipoprotein cholesterol; HDL-C, high-density lipoprotein cholesterol, FFA, free fatty acid.

To further explore the role of hepatic SelS in the metabolic processes, *SelS*^*H-KO*^ mice were generated using the Cre-loxP system. Homozygous *SelS*^*F/F*^ mice were crossed with *Alb-Cre* transgenic mice. Exon 3 was removed by Cre recombinase expression in *SelS*^*H-KO*^ mice, while littermate *SelS*^*F/F*^ mice lacking Cre recombinase expression were used as controls (Fig. 2A and S1A). Western blotting confirmed the deletion of SelS in hepatocytes of *SelS*^*H-KO*^ mice (Fig. 2B). As expected, SelS mRNA and protein expression levels in the liver of *SelS*^*H-KO*^ mice were significantly reduced compared to those in *SelS*^*F/F*^ mice (Figure S1C and S1D). Interestingly, SelS expression levels in subcutaneous WAT (sWAT) and epididymal WAT (eWAT) were increased in *SelS*^*H-KO*^ mice compared with *SelS*^*F/F*^ mice (Fig. S1C and S1D), while similar SelS expression levels were detected in brown adipose tissue (BAT), muscle, heart and kidney tissues of *SelS*^*H-KO*^ and *SelS*^*F/F*^ mice (Fig. S1C and S1D).

To characterize the phenotypes of *SelS*^*H-KO*^ mice under different dietary conditions, mice were fed with either RC or HFD for 20 weeks. The body weight of *SelS*^*H-KO*^ mice was heavier than that of *SelS*^*F/F*^ mice under the same dietary conditions (*P* < 0.05; Fig. 2C). The weight gain of *SelS*^*H-KO*^ mice (15.98 ± 1.32) was greater than that of *SelS*^*F/F*^ mice (14.07 ± 0.39) under RC conditions (*P* < 0.01; Fig. S2A) during development, and the difference was more significant between HFD-fed *SelS*^*H-KO*^ (27.91 ± 0.85) and *SelS*^*F/F*^ mice (22.69 ± 1.68) (*P* < 0.01; Fig. S2A). These results indicate that hepatic SelS deletion promotes the occurrence and development of obesity. As shown in Fig. 2D, the liver weight was similar between *SelS*^*H-KO*^ and *SelS*^*F/F*^ mice fed with RC, whereas the liver weight of *SelS*^*H-KO*^ mice was higher than that of *SelS*^*F/F*^ mice when fed with HFD for 20 weeks (*P* < 0.05). In addition, the heavier body weight of *SelS*^*H-KO*^ mice was mainly due to the increased weight of WAT, including sWAT, eWAT, perirenal WAT and omental WAT (*P* < 0.05). No significant difference was found in the weight of other tissues between *SelS*^*H-KO*^ and *SelS*^*F/F*^ mice (Fig. S2B).

The ratio of liver weight to body weight was similar in *SelS*^*H-KO*^ and *SelS*^*F/F*^ mice (*P* > 0.05; Fig. 2E). As shown in Fig. 2F and G, the color of the liver in *SelS*^*H-KO*^ mice was lighter than that in *SelS*^*F/F*^ mice under RC conditions due to scattered lipid droplet distribution in *SelS*^*H-KO*^ mice liver observed by H&E and Oil Red O staining. The liver of HFD-fed mice became lighter and yellower and had significantly higher levels of lipid deposits than those in RC-fed mice, but the changes were more drastic in *SelS*^*H-KO*^ mice than in *SelS*^*F/F*^ mice. The aggravated hepatosteatosis in *SelS*^*H-KO*^ mice was accompanied by increased serum ALT and AST levels (*P* < 0.05; Fig. 2H). Hepatic SelS deletion also caused dyslipidemia. Compared to those in *SelS*^*F/F*^ mice, the serum levels of TG, TC, LDL-C and FFA were increased in *SelS*^*H-KO*^ mice under the same dietary conditions (*P* < 0.05, Fig. 2I), but the serum HDL-C levels did not differ (*P* > 0.05; Fig. 2I).

### Hepatic-specific deletion of SelS increases hepatic lipid accumulation and ER stress

**Fig. 3:**
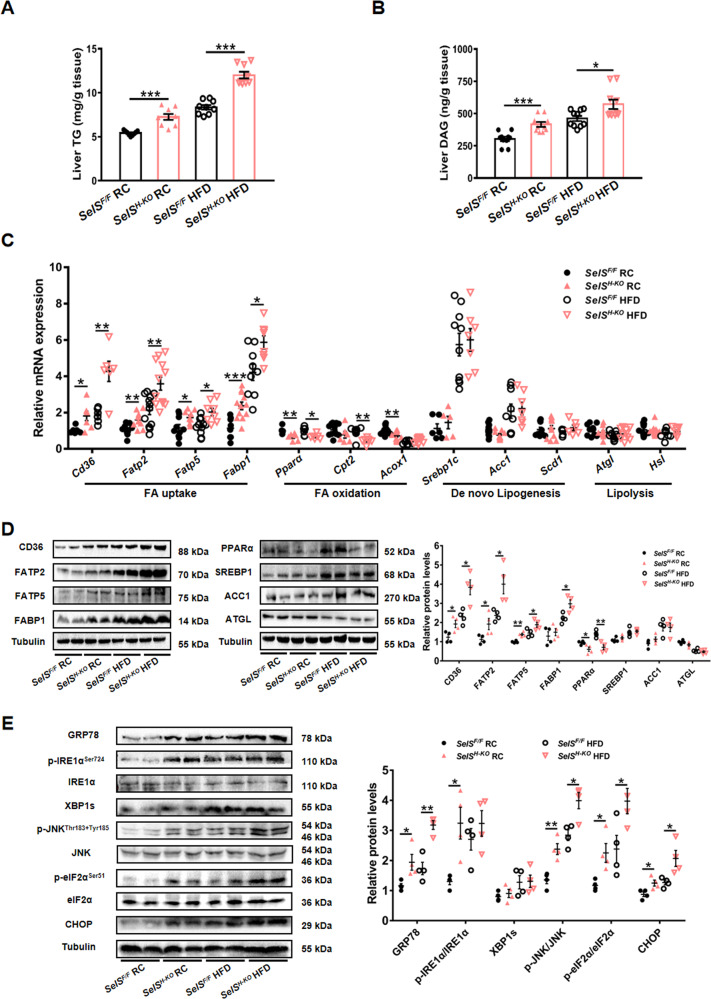
Hepatic-specific deletion of SelS increases hepatic lipid accumulation and ER stress. **A, B** Liver TG (**A**) and DAG (**B**) contents of 24-week-old *SelS*^*H-KO*^ and *SelS*^*F/F*^ mice fed with RC or HFD for 20 weeks (*n* = 9–10 per group). **C** Hepatic mRNA expression of markers involved in fatty acid uptake and oxidation, de novo lipogenesis and lipolysis in the indicated groups (*n* = 5–12 per group). **D** Representative Western blotting images from 2 mice out of 4 for each group and quantified protein levels of markers related to lipid metabolism in the liver of the indicated groups (*n* = 4 per group). **E** Representative Western blotting images from 2 mice out of 4 for each group and quantified protein levels of ER stress markers in the liver of the indicated groups (*n* = 4 per group). All data are presented as the mean ± SEM. *P < 0.05, **P < 0.01, ***P < 0.001. RC, regular chow; HFD, high-fat diet; *SelS*^*H-KO*^, hepatocyte-specific SelS knockout mice; *SelS*^*F/F*^, floxed SelS mice; TG, triglyceride; DAG, diacylglycerol; FA, fatty acid; CD36, cluster of differentiation 36; FATP2/5, fatty acid transport protein 2/5; FABP1, fatty acid binding protein 1; PPARα, peroxisome proliferator-activated receptor α; CPT2, carnitine palmitoyltransferase 2; ACOX1, acetyl-coenzyme A oxidase 1; SREBP1, sterol-regulatory element binding protein 1; ACC1, acetyl-coenzyme A carboxylase 1; SCD1, stearoyl-coenzyme A desaturase 1; ATGL, adipose triglyceride lipase; HSL, hormone-sensitive lipase; GRP78, glucose-regulated protein 78; IRE1α, inositol-requiring enzyme 1α; XBP1s, spliced X-box binding protein-1; JNK, c‐JUN N‐terminal kinase; eIF2α, eukaryotic initiation translation factor 2α; CHOP, CCAAT/enhancer-binding protein homologous protein.

Hepatic TG and DAG contents were significantly increased in *SelS*^*H-KO*^ mice compared with *SelS*^*F/F*^ mice (*P* < 0.05; Fig. 3A and B). We further examined mRNA expression and protein levels of key molecules in hepatic fatty acid uptake, fatty acid oxidation, de novo lipogenesis (DNL) and lipolysis.

As shown in Fig. 3C and 3D, compared to those in *SelS*^*F/F*^ mice, the expression levels of cluster of differentiation 36 (CD36), fatty acid transport protein 2 (FATP2) and fatty acid transport protein 5 (FATP5), which are involved in fatty acid uptake, were markedly increased with statistical significance in *SelS*^*H-KO*^ mice fed with RC or HFD (*P* < 0.05). No significant difference was found in the expression of DNL-related molecules, such as sterol-regulatory element binding protein 1 (SREBP1) and acetyl-coenzyme A carboxylase 1 (ACC1), under the same dietary conditions (*P* > 0.05). Hepatic SelS deletion did not affect the expression of lipolysis-related markers, including adipose triglyceride lipase (ATGL) and hormone-sensitive lipase (HSL) (*P* > 0.05). In contrast, the expression levels of peroxisome proliferator-activated receptor α (PPARα), carnitine palmitoyltransferase 2 (CPT2) and acetyl-coenzyme A oxidase 1 (ACOX1), responsible for fatty acid oxidation, were downregulated in *SelS*^*H-KO*^ mice compared with *SelS*^*F/F*^ mice under RC or HFD conditions (*P* < 0.05). These results suggest that hepatic SelS deletion increases hepatic TG and DAG accumulation via promoting fatty acid uptake and reducing fatty acid oxidation.

In order to investigate the effect of hepatic SelS deletion on ER stress, the expression levels of ER stress markers were analyzed. The expression of GRP78, CCAAT/enhancer-binding protein homologous protein (CHOP), and phosphorylation of eukaryotic translation initiation factor 2α (eIF2α) and c-JUN N-terminal kinase (JNK) were elevated in the liver of *SelS*^*H-KO*^ mice compared with *SelS*^*F/F*^ mice fed with RC or HFD (*P* < 0.05; Fig. 3E). The phosphorylation of IRE1α was significantly elevated in the liver of *SelS*^*H-KO*^ mice under RC conditions (*P* < 0.05; Fig. 3E), though hepatic SelS deletion did not alter IRE1α phosphorylation under HFD conditions (*P* > 0.05; Fig. 3E) or the expression levels of spliced X-box binding protein-1 (XBP1s) under RC or HFD conditions (*P* > 0.05; Fig. 3E).

### Hepatic-specific deletion of SelS contributes to IR, impaired glucose metabolism and promotes hepatic PKCɛ activation

**Fig. 4:**
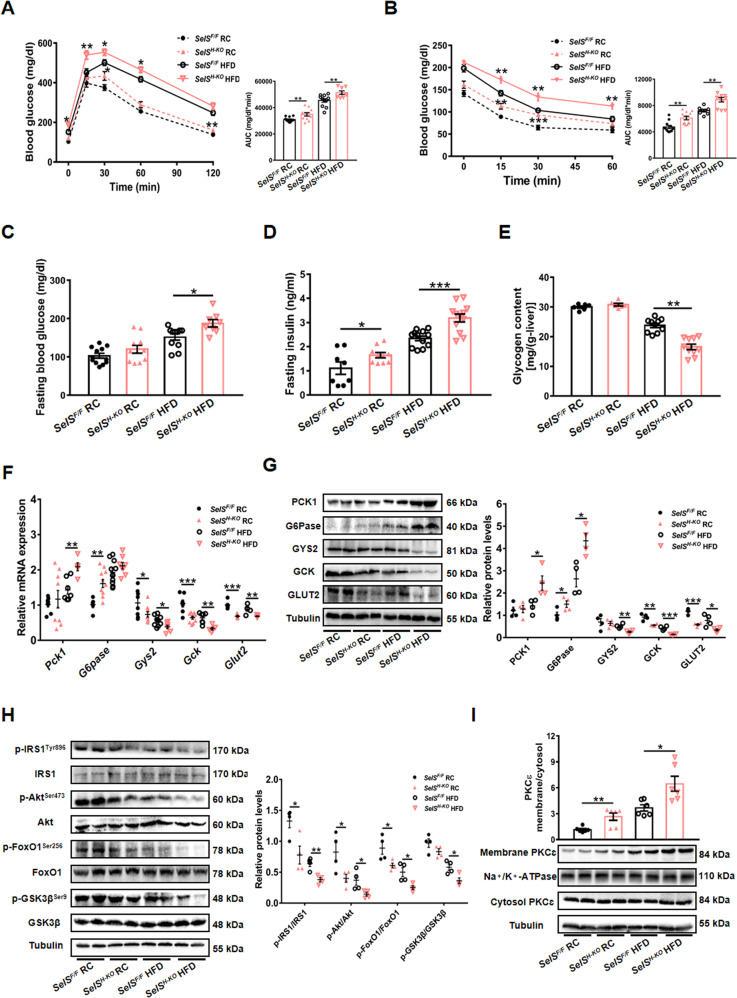
Hepatic-specific deletion of SelS contributes to IR, impaired glucose metabolism and promotes hepatic PKCɛ activation. **A** Glucose tolerance test (GTT) and the area under curve (AUC) during GTT of 24-week-old *SelS*^*H-KO*^ and *SelS*^*F/F*^ mice fed with RC or HFD for 20 weeks (*n* = 8–12 per group). **B** Insulin tolerance test (ITT) and the AUC during ITT in the indicated groups (*n* = 8–12 per group). **C** Fasting blood glucose levels in the indicated groups (*n* = 8–12 per group). **D** Fasting insulin levels in the indicated groups (*n* = 8–12 per group). **E** Hepatic glycogen content in the indicated groups (*n* = 6–10 per group). F Relative mRNA expression of glucose metabolism-related markers in the liver of the indicated groups (*n* = 5–10 per group). **G** Representative Western blotting images from 2 mice out of 4 for each group and quantified protein levels of glucose metabolism-related markers in the liver of the indicated groups (*n* = 4 per group). **H** The phosphorylation and total protein levels of IRS1, Akt, FoxO1, and GSK3β in the liver of the indicated groups (*n* = 4 per group). Representative Western blotting images from 2 mice out of 4 for each group are shown. **I** Membrane/cytosol ratio of hepatic PKCɛ protein levels normalized with housekeeping proteins Na^+^/K^+^-ATPase and Tubulin in the indicated groups (*n* = 6 per group). Representative images from 2 mice out of 6 for each group are shown. All data are presented as the mean ± SEM. *P < 0.05, **P < 0.01, ***P < 0.001. RC, regular chow; HFD, high-fat diet; *SelS*^*H-KO*^, hepatocyte-specific SelS knockout mice; *SelS*^*F/F*^, floxed SelS mice; PCK1, phosphoenolpyruvate carboxykinase 1; G6Pase, glucose-6-phosphatase; GYS2, glycogen synthase 2; GCK, glucokinase; GLUT2, glucose transporter 2; IRS1, insulin receptor substrate 1; Akt/PKB, protein kinase B; FoxO1, forkhead box protein O1; GSK3β, glycogen synthase kinase 3β; PKCɛ, protein kinase Cɛ.

To evaluate the effect of hepatic SelS deletion on IR and glucose metabolism, GTT and ITT were performed. Blood glucose levels were higher by GTT and ITT in *SelS*^*H-KO*^ mice than in *SelS*^*F/F*^ mice fed with RC or HFD (*P* < 0.05; Fig. 4A and B), indicating that hepatic SelS deletion diminishes glucose tolerance and insulin sensitivity. Hepatic SelS deficiency increased fasting blood glucose levels under HFD conditions (*P* < 0.05; Fig. 4C). The fasting insulin levels of *SelS*^*H-KO*^ mice were significantly increased under the same dietary conditions (*P* < 0.05; Fig. 4D). Additionally, *SelS*^*H-KO*^ mice exhibited lower hepatic glycogen content when fed with HFD for 20 weeks (*P* < 0.01; Fig. 4E).

Next, the influence of hepatic SelS deletion on the expression of hepatic glucose metabolism-related genes and proteins was examined. As shown in Fig. 4F and G, the expression levels of glucose-6-phosphatase (G6Pase) and phosphoenolpyruvate carboxykinase 1 (PCK1), the key enzymes in gluconeogenesis, were elevated in *SelS*^*H-KO*^ mice liver (*P* < 0.05), whereas hepatic SelS deficiency inhibited the expression of glycogen synthase 2 (GYS2), the enzyme promoting glycogen synthesis in the liver (*P* < 0.05). Glucokinase (GCK) catalyzes one of the regulated steps of glycolysis and hepatic glycogen synthesis [31]. Glucose transporter 2 (GLUT2) is responsible for glucose uptake into hepatocytes [32]. *SelS*^*H-KO*^ mice showed notably lower expression levels of GCK and GLUT2 under the same dietary conditions (*P* < 0.05; Fig. 4F and G).

In parallel, we found that hepatic SelS deletion inhibited insulin signaling, as reflected by a reduction in phosphorylation of IRS1 and Akt (*P* < 0.05; Fig. 4H). The reduced Akt activation led to decreased phosphorylation of forkhead box protein O1 (FoxO1) at serine (Ser)^256^ and glycogen synthase kinase 3β (GSK3β) at Ser^9^ in the *SelS*^*H-KO*^ mice liver (*P* < 0.05; Fig. 4H). Previous studies have shown that hepatic PKCɛ activation participants in the pathogenesis of IR [33]. We found a significant increase in PKCɛ translocation to the plasma membrane in *SelS*^*H-KO*^ mice (*P* < 0.05; Fig. 4I).

### SelS protects mouse primary hepatocytes from hepatosteatosis in vitro

**Fig. 5:**
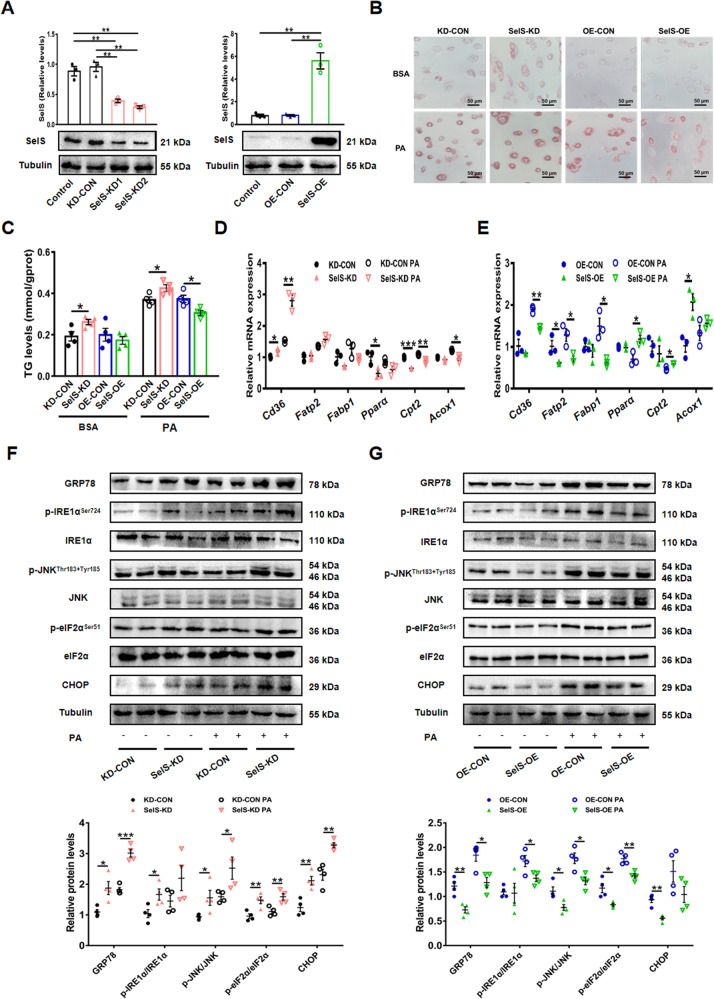
SelS protects mouse primary hepatocytes from hepatosteatosis in vitro. **A** Representative Western blotting images from one sample out of 3 for each group and quantified protein levels of SelS after the primary hepatocytes isolated from wild-type mice were infected with adenoviruses for 24 h (*n* = 3 per group). **B** After infection, Oil Red O staining were performed in primary hepatocytes treated with 0.5 mM PA or BSA for 24 h. Scale bar, 50 μm. **C** TG contents in the primary hepatocytes (*n* = 4 per group). **D**, **E** Relative mRNA expression of markers involved in fatty acid uptake and oxidation in SelS-KD and KD-CON (**D**), SelS-OE and OE-CON (**E**) primary hepatocytes treated with 0.5 mM PA or BSA for 24 h (*n* = 3 per group). **F**, **G** Representative Western blotting images from 2 samples out of 4 for each group and quantified protein levels of ER stress markers in SelS-KD and KD-CON (**F**), SelS-OE and OE-CON (**G**) primary hepatocytes treated with 0.5 mM PA or BSA for 24 h (*n* = 4 per group). All data are presented as the mean ± SEM. *P < 0.05, **P < 0.01, ***P < 0.001. SelS-KD/KD-CON, SelS knockdown and the corresponding control; SelS-OE/OE-CON, SelS overexpression and the corresponding control; TG, triglyceride; CD36, cluster of differentiation 36; FATP2, fatty acid transport protein 2; FABP1, fatty acid binding protein 1; PPARα, peroxisome proliferator-activated receptor α; CPT2, carnitine palmitoyltransferase 2; ACOX1, acetyl-coenzyme A oxidase 1; GRP78, glucose-regulated protein 78; IRE1α, inositol-requiring enzyme 1α; JNK, c‐JUN N‐terminal kinase; eIF2α, eukaryotic initiation translation factor 2α; CHOP, CCAAT/enhancer-binding protein homologous protein.

At the cellular level, we knocked down SelS in mouse primary hepatocytes using two different SelS shRNA (SelS-KD1, SelS-KD2) delivered by adenoviruses. On the other hand, the mouse primary hepatocytes were infected with a SelS expressing adenovirus (SelS-OE) for SelS overexpression. Western blotting analysis showed an obvious decrease of SelS expression in SelS-KD cells (*P* < 0.01; Fig. 5A) and a robust SelS overexpression in SelS-OE cells (*P* < 0.01; Fig. 5A). Lipid accumulation and TG levels were increased in SelS-KD hepatocytes compared to those in the KD-CON hepatocytes with or without PA treatment (*P* < 0.05; Fig. 5B and C). On the contrary, compared with OE-CON hepatocytes, PA-induced lipid deposition and TG accumulation were attenuated in SelS-OE hepatocytes (*P* < 0.05; Fig. 5B and C). SelS knockdown in hepatocytes increased fatty acid uptake via elevated *Cd36* mRNA expression, and upregulated the protein expression levels of ER stress markers, including GRP78, CHOP, and IRE1α, JNK and eIF2α phosphorylation (*P* < 0.05; Fig. 5D and F). The expression levels of key markers in fatty acid uptake and ER stress were decreased in SelS-OE cells compared to OE-CON cells (*P* < 0.05; Fig. 5E and G). Besides, mRNA expression levels of *Pparα*, *Cpt2* and *Acox1* participating in fatty acid oxidation were downregulated in SelS-KD cells, but were enhanced in SelS-OE cells compared to the control, respectively (*P* < 0.05; Fig. 5D and E). Thereby, consistent with studies in *SelS*^*H-KO*^ mice, hepatosteatosis and ER stress were enhanced in SelS-KD hepatocytes, whereas both were suppressed by SelS overexpression in hepatocytes. Our data reveal a significantly protective role of SelS against hepatosteatosis and ER stress.

### SelS promotes hepatic insulin signaling via decreased PKCɛ activation in vitro

**Fig. 6:**
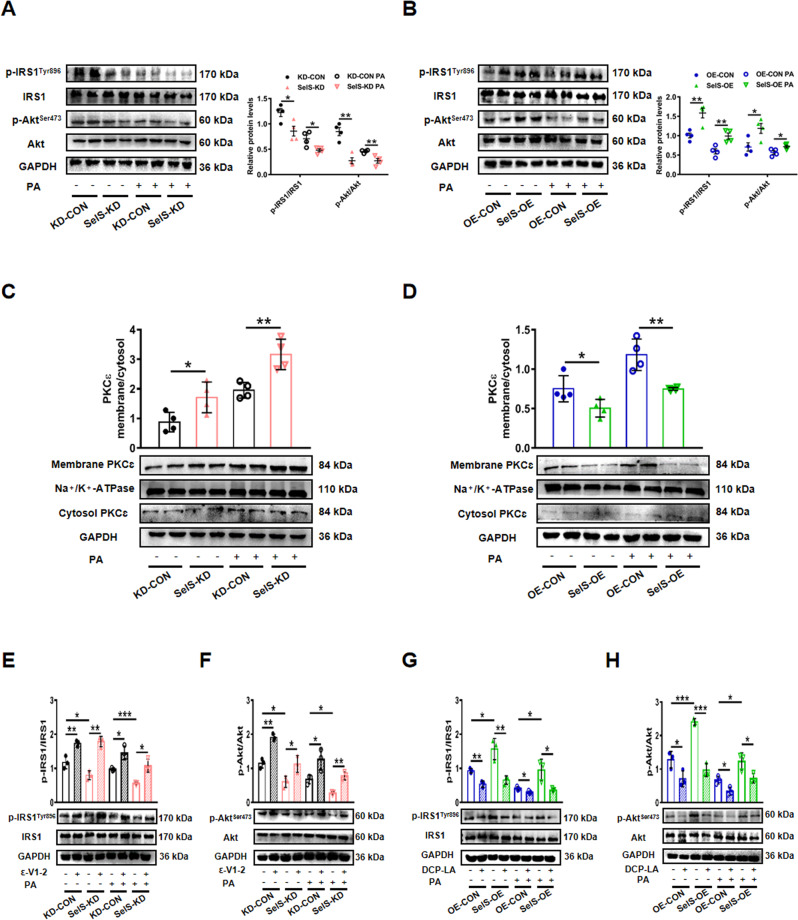
SelS promotes hepatic insulin signaling via decreased PKCɛ activation in vitro. **A**, **B** The phosphorylation and total protein levels of IRS1 and Akt in SelS-KD and KD-CON (**A**), SelS-OE and OE-CON (**B**) primary hepatocytes treated with 0.5 mM PA or BSA for 24 h (*n* = 4 per group). Representative Western blotting images from 2 samples out of 4 for each group are shown. **C**, **D** Membrane/cytosol ratio of hepatic PKCɛ protein levels normalized with housekeeping proteins Na^+^/K^+^-ATPase and GAPDH in SelS-KD and KD-CON (**C**), SelS-OE and OE-CON (**D**) primary hepatocytes treated with 0.5 mM PA or BSA for 24 h (*n* = 4 per group). Representative images from two samples out of 4 for each group are shown. **E**–**H** After infection, the primary hepatocytes were preincubated with PKCɛ inhibitor (ε-V1-2, 1 μM) in SelS-KD and KD-CON groups (**E**, **F**) or PKCɛ activator (DCP-LA, 100 nM) in SelS-OE and OE-CON groups (**G**, **H**) for 1 h before stimulated with 0.5 mM PA or BSA for 24 h. The phosphorylation and total protein levels of IRS1 and Akt were examined by Western blotting in the indicated groups (*n* = 3 per group). Representative images from one sample out of 3 for each group are shown. All data are presented as the mean ± SEM. *P < 0.05, **P < 0.01, ***P < 0.001. SelS-KD/KD-CON, SelS knockdown and the corresponding control; SelS-OE/OE-CON, SelS overexpression and the corresponding control; IRS1, insulin receptor substrate 1; Akt/PKB, protein kinase B; PKCɛ, protein kinase Cɛ.

We next tested whether SelS affected insulin signaling in vitro. The phosphorylation levels of IRS1 and Akt were decreased in SelS-KD cells compared with KD-CON cells (*P* < 0.05; Fig. 6A), while increased IRS1 and Akt phosphorylation levels were observed in SelS-OE cells compared to those in OE-CON cells with or without PA treatment (*P* < 0.05; Fig. 6B). In agreement with the results in vivo, compared to KD-CON cells, PKCɛ activation was elevated in SelS-KD cells, as reflected by enhanced PKCɛ translocation to plasma membrane (*P* < 0.05; Fig. 6C). Meanwhile, PKCɛ activation was reduced in SelS-OE cells (*P* < 0.05; Fig. 6D). Subsequently, PKCɛ inhibitor ε-V1-2 and activator DCP-LA were used to verify whether PKCɛ was involved in the effect of SelS on insulin signaling. We found elevated phosphorylation levels of IRS1 and Akt in SelS-KD cells after treatment with ε-V1-2 (*P* < 0.05; Fig. 6E and F). Moreover, the phosphorylation levels of IRS1 and Akt were decreased in SelS-OE cells pretreated by PKCɛ activator DCP-LA (*P* < 0.05; Fig. 6G and H). Collectively, these data suggest that SelS may promote hepatic insulin signaling through inhibiting hepatic PKCɛ activation.

### Hepatic-specific deletion of SelS decreases the production of hepatokine FGF21 and adipokine adiponectin, and increases adipose tissue size

**Fig. 7:**
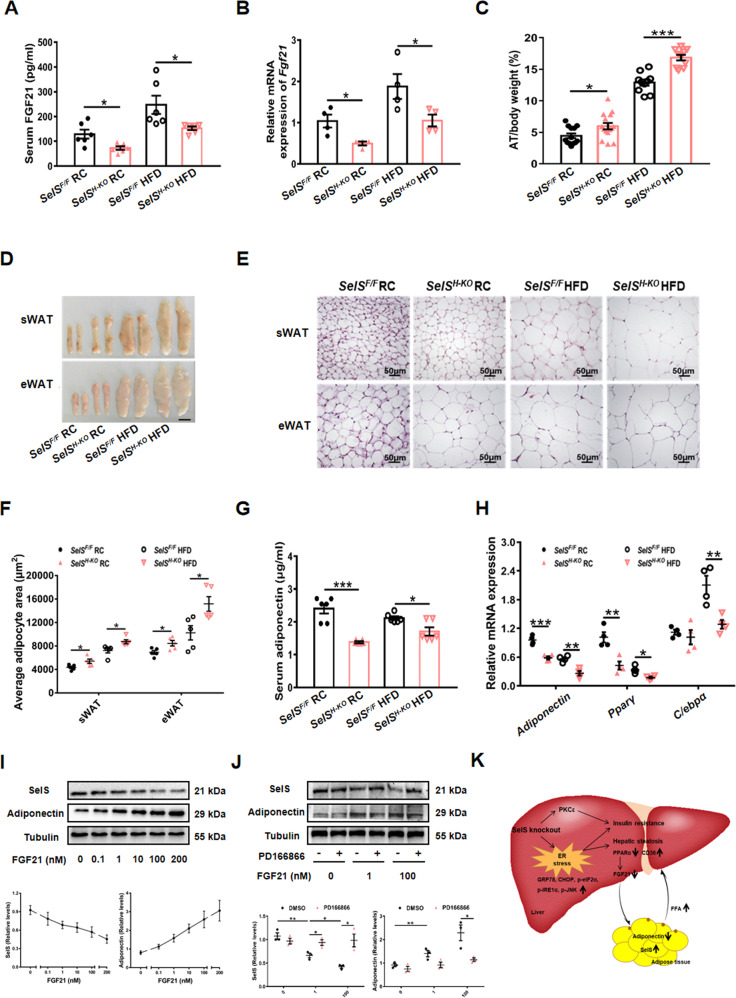
Hepatic-specific deletion of SelS decreases the production of hepatokine FGF21 and adipokine adiponectin and increases adipose tissue size. **A**, **B** Serum FGF21 concentrations (**A**) and *Fgf21* mRNA expression (**B**) in the liver of 24-week-old *SelS*^*H-KO*^ and *SelS*^*F/F*^ mice fed with RC or HFD for 20 weeks (*n* = 6 or 4 per group). **C** AT weight/body weight ratio of the indicated groups (*n* = 10–15 per group). **D** Representative images of sWAT and eWAT isolated from mice of the indicated groups. Scale bar, 1 cm. **E** H&E staining of sWAT and eWAT in the indicated groups. Scale bar, 50 μm. **F** Quantification of adipocyte area of sWAT and eWAT in the indicated groups (*n* = 5 per group). **G** Serum adiponectin concentrations in the indicated groups (*n* = 6 per group). **H** Relative mRNA expression of *Adiponectin*, *Pparγ* and *C/ebpα* in sWAT of the indicated groups (*n* = 4 per group). **I** Western blotting analysis of SelS and adiponectin protein levels in the mature mouse adipocytes, which were treated with different concentrations of recombinant mouse FGF21 for 24 h. The experiments are repeated for five times. **J** Representative Western blotting images from one sample out of 3 for each group and quantified protein levels of SelS and adiponectin after mature mouse adipocytes were stimulated with FGF receptor-1 (FGFR1) inhibitor (PD166866, 10 μM) or DMSO for 1 h, and subsequently treated with different concentrations of recombinant mouse FGF21 for 24 h (*n* = 3 per group). **K** Schematic illustration of hypothesized mechanisms. All data are presented as the mean ± SEM. *P < 0.05, **P < 0.01, ***P < 0.001. RC, regular chow; HFD, high-fat diet; *SelS*^*H-KO*^, hepatocyte-specific SelS knockout mice; *SelS*^*F/F*^, floxed SelS mice; FGF21, fibroblast growth factor 21; AT, adipose tissue; sWAT, subcutaneous white adipose tissue; eWAT, epididymal white adipose tissue; PPARγ, peroxisome proliferator-activated receptor γ; C/EBPα, CCAAT/enhancer-binding protein α; PKCɛ, protein kinase Cɛ; GRP78, glucose-regulated protein 78; eIF2α, eukaryotic translation initiation factor 2α; CHOP, CCAAT/enhancer-binding protein homologous protein; IRE1α, inositol-requiring enzyme 1α; JNK, c‐JUN N‐terminal kinase; CD36, cluster of differentiation 36; PPARα, peroxisome proliferator-activated receptor α; FFA, free fatty acid.

FGF21 is an endocrine hepatokine produced predominantly in the liver [34]. FGF21 exerts its effects on regulating glucose and lipid metabolism by binding to FGF receptors and the co-receptor β-Klotho, which are highly expressed in adipocytes [35]. Serum FGF21 levels and hepatic *Fgf21* mRNA expression were decreased in *SelS*^*H-KO*^ mice compared with *SelS*^*F/F*^ mice under the same dietary conditions (*P* < 0.05; Fig. 7A and B). Another hepatokine fetuin-A has also been suggested to mediate pathophysiological processes of metabolic diseases, of which circulating levels and hepatic mRNA expression were not altered in *SelS*^*H-KO*^ mice (Fig. S3A and S3B). The ratio of adipose tissue weight to body weight was higher in *SelS*^*H-KO*^ mice than in *SelS*^*F/F*^ mice under the same dietary conditions (*P* < 0.05; Fig. 7C). sWAT and eWAT of *SelS*^*H-KO*^ mice were larger due to an increase in adipocyte size (*P* < 0.05; Fig. 7D–F). In addition, we measured adiponectin levels and adiponectin production related genes expression in WAT. Compared to those in *SelS*^*F/F*^ mice, the serum adiponectin concentration and adipose tissue *Adiponectin* mRNA expression were reduced in *SelS*^*H-KO*^ mice under the same dietary conditions (*P* < 0.05; Fig. 7G and H). The expression of *Pparγ* and CCAAT/enhancer-binding protein α (*C/ebpα*), the transcription factors that regulate adiponectin expression, was markedly decreased in sWAT of *SelS*^*H-KO*^ mice (*P* < 0.05; Fig. 7H). Furthermore, recombinant mouse FGF21 treatment caused a significant elevation of adiponectin expression in a dose-dependent manner and a progressive decrease of SelS protein levels in mouse adipocytes (Fig. 7I). Conversely, these effects were impaired in adipocytes pretreated by FGFR1 inhibitor PD166866 (*P* < 0.05; Fig. 7J).

## Discussion

Liver acts as an essential organ in glucose and lipid metabolism responsible for maintaining energy homeostasis. SelS is highly expressed in the liver. Previous studies have shown that SelS is involved in the regulation of glycolipid metabolism [7, 8, 10–13]. Here we found a reduction in hepatic SelS expression in HFD-fed mice and *db/db* mice in vivo, and decreased SelS expression levels in the PA-induced primary hepatocytes in vitro, indicating that hepatic SelS performs vital regulatory functions in metabolic processes. These findings were in accordance with further studies in *SelS*^*H-KO*^ mice. We reveal that the loss of hepatic SelS leads to obesity, hepatic steatosis, IR and disturbed glucose homeostasis.

Hepatic TG synthesis consists of two major pathways, known as fatty acid uptake and DNL. Our study showed that hepatic fatty acid uptake was markedly elevated in *SelS*^*H-KO*^ mice. In patients with NAFLD, 59% of TG in the liver is originated from circulating FFA, 26% from DNL, and 15% from diet [36]. Since elevated serum FFA level was found in *SelS*^*H-KO*^ mice compared with *SelS*^*F/F*^ mice and DNL was not affected in the liver of *SelS*^*H-KO*^ mice, hepatic SelS deletion more likely promoted hepatic lipid acquisition through increased FFA uptake. Impaired fatty acid oxidation also contributes to hepatic steatosis. PPARα participates in fatty acid oxidation by regulating the transcription of fatty acid oxidation-related genes to reverse hepatic steatosis [37]. The results provide the evidence that hepatic SelS deletion promotes hepatic lipid accumulation by enhancing fatty acid uptake and reducing fatty acid oxidation. The phenotype is reinforced in primary hepatocytes, suggesting that hepatic SelS exhibits an inhibitory role in hepatosteatosis.

The ER in hepatocytes is a critical organelle for hepatic metabolic adaptation. Chronic ER stress affects major pathways of lipid metabolism by the alteration of fatty acid uptake, DNL, very low density lipoprotein secretion and fatty acid oxidation, which contributes to hepatic steatosis [17, 18, 38]. In the liver, ER stress causes hyperactivation of JNK, leading to decreased tyrosine phosphorylation of IRS-1 and subsequent IR, which is also a risk factor for the progression of NAFLD [20, 39]. Consistent with previous studies identifying that SelS silencing increased ER stress markers expression [24, 27], while SelS overexpression protected several cell lines from ER stress injury [26–28], we further confirmed ER stress was increased in *SelS*^*H-KO*^ mice and SelS-KD hepatocytes, but suppressed in SelS-OE hepatocytes. Thus, we speculate that the retro-translocation of misfolded or unfolded proteins in the hepatocytes facilitated by SelS binding to ERAD components may be blocked due to the loss of hepatic SelS, resulting in chronic ER stress. In contrast, upregulation of hepatic SelS protects hepatocytes from hepatosteatosis via attenuating ER stress.

Ectopic lipid accumulation within the liver is often accompanied with hepatic IR [3, 40, 41]. PKCε is a novel PKC isoform that is typically activated in the liver during HFD feeding [42]. PKCε phosphorylates insulin receptor threonine^1160^, resulting in inhibition of insulin receptor kinase activity and insulin signaling [43]. Previous studies have shown that the molecular structure of SelS contains 4 PKC phosphorylation sites [7], as well as selenocystine that readily inactivates the kinase activity of PKC [44, 45]. Our data suggest that hepatic SelS regulates insulin action partially through PKCε by directly pharmacological manipulation of PKCε in SelS overexpression and knockdown primary hepatocytes. On the other hand, accumulating evidence showed that in NAFLD, DAG accumulation in hepatocytes is involved in the pathogenesis of hepatic IR through PKCε activation [30, 40, 46]. The current study found hepatic deficiency of SelS caused and exacerbated HFD-induced hepatosteatosis, hence, increased DAG levels in *SelS*^*H-KO*^ mice presumably mediated PKCε activation and inhibited hepatic insulin pathway. Overall, both enhanced ER stress and PKCε overactivation caused hepatic IR in *SelS*^*H-KO*^ mice, resulting in suppression of hepatic glycogen synthesis and glucose uptake as well as stimulation of hepatic gluconeogenesis, which led to decreased glucose tolerance and insulin sensitivity. Consistent with our results, Walder et al. [7] found that compared to *Psammomys obesus* (*P. obesus*) with normal glucose tolerance, hepatic SelS expression was reduced in *P. obesus* with impaired glucose tolerance and T2DM. SelS gene expression in the liver was inversely correlated with circulating glucose and insulin levels in *P. obesus* [7]. However, our results were in contrast to those of a study identifying that SelS overexpression in hepatoma H4IIE cells decreased insulin sensitivity through reductions in insulin-stimulated glucose utilization and suppression of hepatic glucose production without affecting the phosphorylation of insulin receptor and IRS-1 (Ref. [13]). The inconsistent results may be due to the different experimental conditions of in vivo and in vitro studies. Nonetheless, we demonstrate that SelS expression in the liver suppresses hepatic IR to improve glucose metabolism.

Remarkably, we found hepatic SelS deletion affected the inter-organ communication. FGF21 is a metabolic regulatory factor primarily secreted from liver and has profound effects on multiple tissues. FGF21 can be induced by PPARα in the liver during starvation and the use of PPARα agonists, indicating FGF21 is the downstream target of PPARα [47]. Studies have shown increased FGF21 in obesity and NAFLD [48, 49], whereas pharmacological administration of FGF21 is beneficial to body weight loss, alleviation of fatty liver, dyslipidemia and hyperglycemia [50–52]. Accumulating evidence showed a positive correlation between FGF21 and adiponectin [53, 54]. FGF21-adiponectin axis plays a role in regulating glycolipid metabolism, in which FGF21 induces adiponectin derived from adipose tissue and in turn, adiponectin reduces hepatic lipid accumulation and IR [55, 56]. The present study suggest that FGF21-adiponectin axis is inhibited in *SelS*^*H-KO*^ mice, which exacerbates hepatic metabolic disorders. Upregulation of FGF21 under HFD conditions may be a protective effect in response to significant disturbed energy homeostasis due to excessive nutrients uptake. In addition, our data tested the hypothesis that reduced FGF21 secretion from hepatocytes may be a key factor for the explanation of elevated adipose tissue SelS expression in *SelS*^*H-KO*^ mice through the use of FGFR1 inhibitor PD166866 in adipocytes. We found PD166866 blocked the inhibitory effect of FGF21 on SelS expression in adipocytes. Our previous studies revealed SelS promoted adipocytes survival and adipogenesis to participate in the occurrence of obesity [11, 12]. Increased SelS expression in the WAT aggravated fat expansion. Subsequently, lipolysis was markedly enhanced when TG accumulation overloaded, which increased the delivery of FFAs to the liver and further promoted ectopic lipid deposition in the liver (Fig. S4).

The present study showed hepatic SelS deletion resulted in increased body weight and abnormal glycolipid metabolism. However, Addinsall et al. [57] reported that whole-body SelS deletion had no obvious effect on body weight, fat mass and lean mass by generation of global SelS-deleted (GKO) mice. It is noteworthy that Addinsall et al. [57] only checked the body weight in mice up to 12-week old. Their study focused on muscle functions and may ignore the defects in the later developmental stages of the GKO mice. Besides, we reported SelS expression in adipose tissue promoted the pathogenesis and progression of obesity and IR [9, 11]. Therefore, the lack effect on body weight and metabolism in GKO mice may also due to the reduction of adipose tissue SelS expression, which can blunt the disadvantages of decreased SelS in the liver for glycolipid metabolism. The current study clarifying the effect of SelS on hepatosteatosis and IR mainly focused on male mice, since female mice are hormonally protected and less sensitive to obesity-associated metabolic disorders [58]. It is likely that the metabolic phenotype and mechanism are similar between female and male SelS^*H-KO*^ mice, although certain characteristics might be more profound in males or male-specific.

In summary, using *SelS*^*H-KO*^ mice, we first found hepatic SelS deficiency increases ER stress, resulting in hepatic lipid accumulation and impaired insulin signaling, while SelS overexpression protects primary hepatocytes from these metabolic disorders. The present study also revealed SelS functions partially through inhibiting PKCε activation against insulin resistance. Moreover, we proposed SelS may mediate the liver-adipose tissue crosstalk to maintain the homeostasis of glycolipid metabolism by regulating FGF21-adiponectin axis. Our data suggest that SelS may represent a novel target for the prevention and treatment of NAFLD and T2DM (Fig. 7K).

## Supplementary information

aj-checklist

Figure legends

Figure S1

Figure S2

Figure S3

Figure S4

Tables

Uncut Western blots

## Data Availability

All data required to support the findings of this study are included in this published article and supplementary materials.
