# Supplementary material for: Hepatic deficiency of selenoprotein S exacerbates hepatic steatosis and insulin resistance
Source: Cell Death Dis. 2022 Mar 28;13(3):275. doi: 10.1038/s41419-022-04716-w (PMC8960781; doi:10.1038/s41419-022-04716-w)
Supplement: Supplementary file 2 — Figure legends [file 41419_2022_4716_MOESM2_ESM.docx]

**Figure legends**


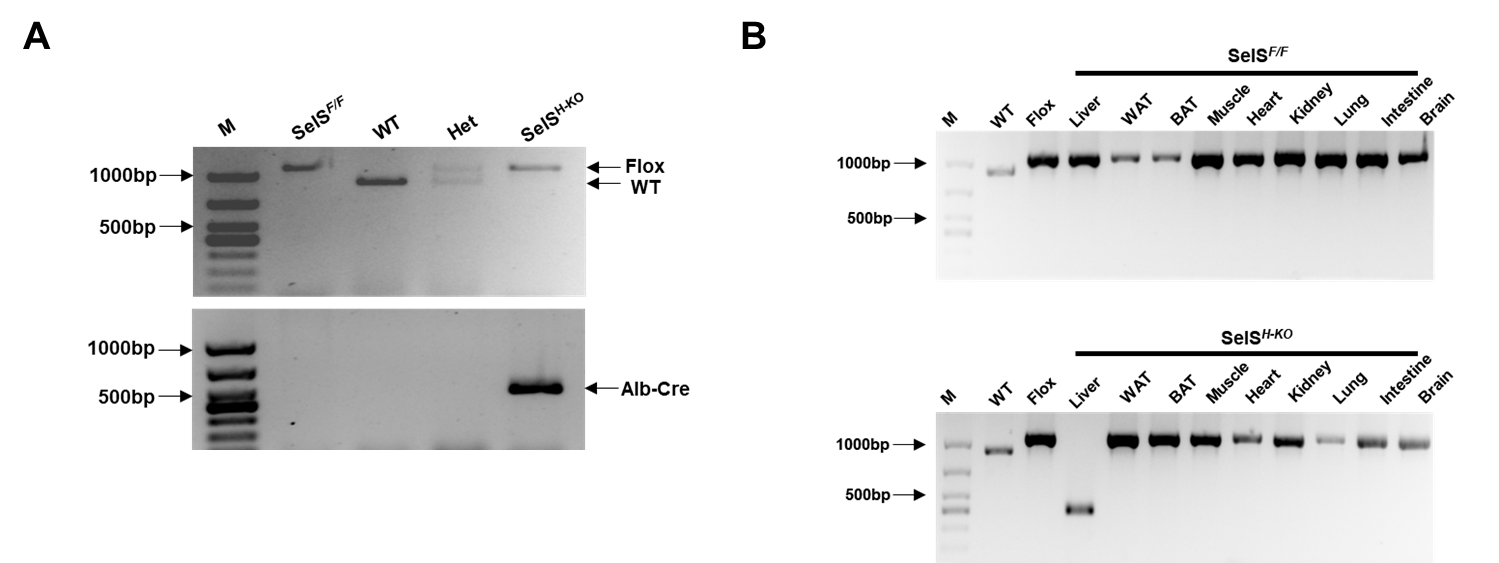
**Figure S1.** **Genotyping of** ***SelS^H-KO^* and *SelS^F/F^* mice. A** PCR genotyping of *SelS^H-KO^* and *SelS^F/F^* mice. **B** Confirmation of hepatic-specific deletion of SelS by PCR in different tissues. Note that a smaller deletion band was detected only in the liver of *SelS^H-KO^* mice due to the removal of exon 3 of SelS upon Cre recombinase expression. **C** *SelS* mRNA expression in various tissues of *SelS^H-KO^* and *SelS^F/F^* mice (n=7-12 per group). **D** Western blot analysis of SelS protein expression levels in various tissues of *SelS^H-KO^* and *SelS^F/F^* mice (n=3 per group). Representative images from one mouse out of 3 for each group are shown. All data are presented as the mean ± SEM. ****P*<0.001. SelS, selenoprotein S; WT, wild-type; Het, heterozygote; *Alb-Cre*, *Albumin-Cre*; *SelS^H-KO^*, hepatocyte-specific SelS knockout mice; *SelS^F/F^*, floxed SelS mice; M, marker; WAT, white adipose tissue; BAT, brown adipose tissue; sWAT, subcutaneous white adipose tissue; eWAT, epididymal white adipose tissue.

**Figure S2.** **Body weight gain and organ weight of *SelS^H-KO^* and *SelS^F/F^* mice. A** Body weight gain (24 weeks versus 4 weeks) of RC- or HFD-fed *SelS^H-KO^* and *SelS^F/F^* mice (n=10-12 per group). **B** Organ weight of 24-week-old *SelS^H-KO^* and *SelS^F/F^* mice fed with RC or HFD for 20 weeks (n=10-15 per group). All data are presented as the mean ± SEM. ***P*<0.01. RC, regular chow; HFD, high-fat diet; *SelS^H-KO^*, hepatocyte-specific SelS knockout mice; *SelS^F/F^*, floxed SelS mice.

**Figure S3.** **Serum fetuin-A concentration** **and hepatic fetuin-A expression of *SelS^H-KO^* and *SelS^F/F^* mice.** **A, B** Serum fetuin-A concentration (A) and *fetuin-A* mRNA expression (B) in the liver of 24-week-old *SelS^H-KO^* and *SelS^F/F^* mice fed with RC or HFD for 20 weeks (n=6 or 4 per group). All data are presented as the mean ± SEM. RC, regular chow; HFD, high-fat diet; *SelS^H-KO^*, hepatocyte-specific SelS knockout mice; *SelS^F/F^*, floxed SelS mice.

**Figure S4. Lipid metabolism in sWAT and eWAT of *SelS^H-KO^* and *SelS^F/F^* mice.**

**A, B** Relative mRNA expression of markers related to lipogenesis and lipolysis in sWAT (A) and eWAT (B) in 24-week-old *SelS^H-KO^* and *SelS^F/F^* mice fed with RC or HFD for 20 weeks (n=5-10 per group). All data are presented as the mean ± SEM. ***P*<0.01, ****P*<0.001. RC, regular chow; HFD, high-fat diet; *SelS^H-KO^*, hepatocyte-specific SelS knockout mice; *SelS^F/F^*, floxed SelS mice; sWAT, subcutaneous white adipose tissue; eWAT, epididymal white adipose tissue; ACC1, acetyl-coenzyme A carboxylase 1; SCD1, stearoyl-coenzyme A desaturase 1; ATGL, adipose triglyceride lipase; HSL, hormone-sensitive lipase; MGL, monoacylglycerol lipase.
