# Supplementary figures and images for: Hepatic deficiency of selenoprotein S exacerbates hepatic steatosis and insulin resistance
Source: Cell Death Dis. 2022 Mar 28;13(3):275. doi: 10.1038/s41419-022-04716-w (PMC8960781; doi:10.1038/s41419-022-04716-w)

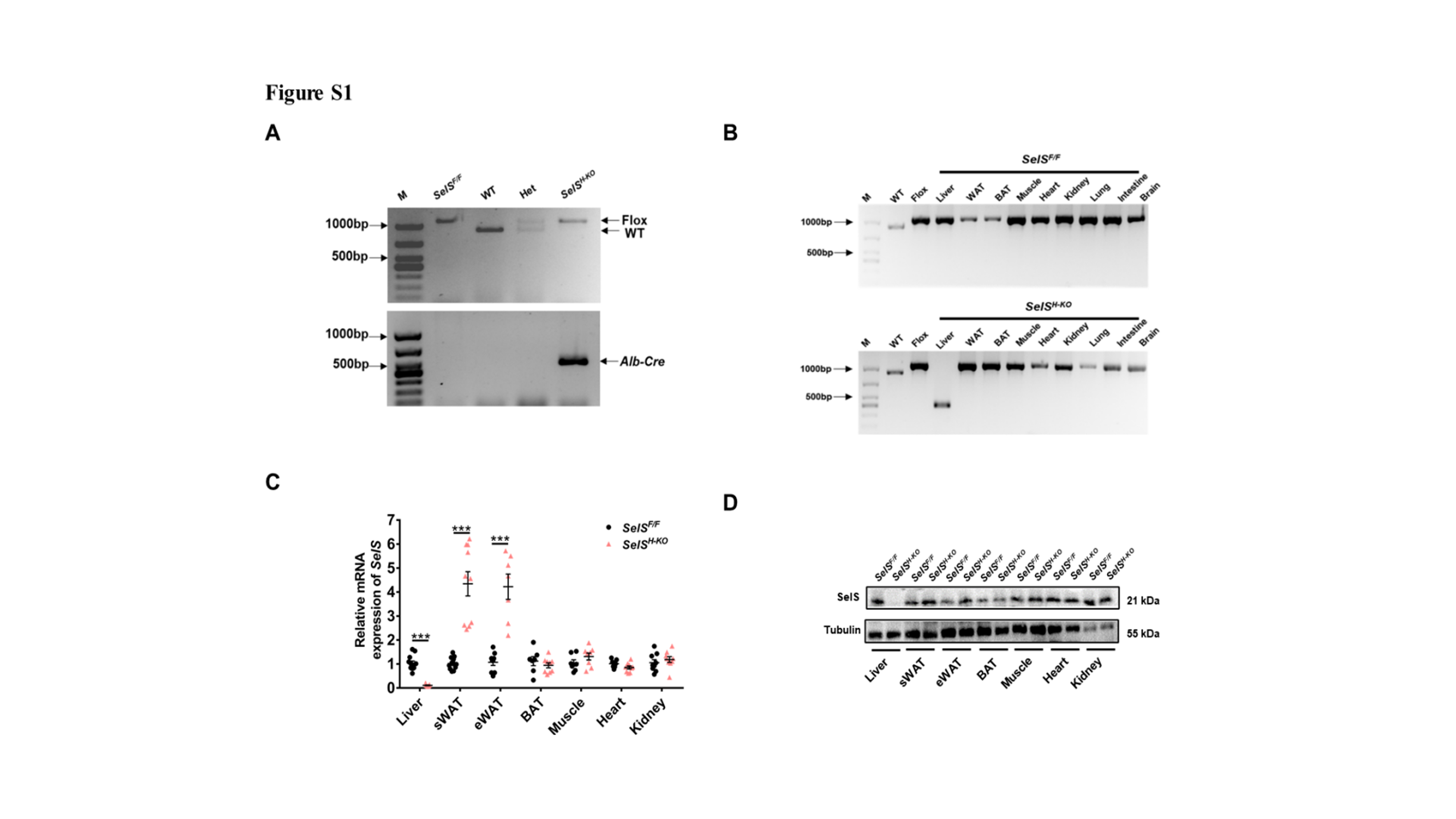

Supplement: Supplementary file 3 — Figure S1 [file 41419_2022_4716_MOESM3_ESM.tif]

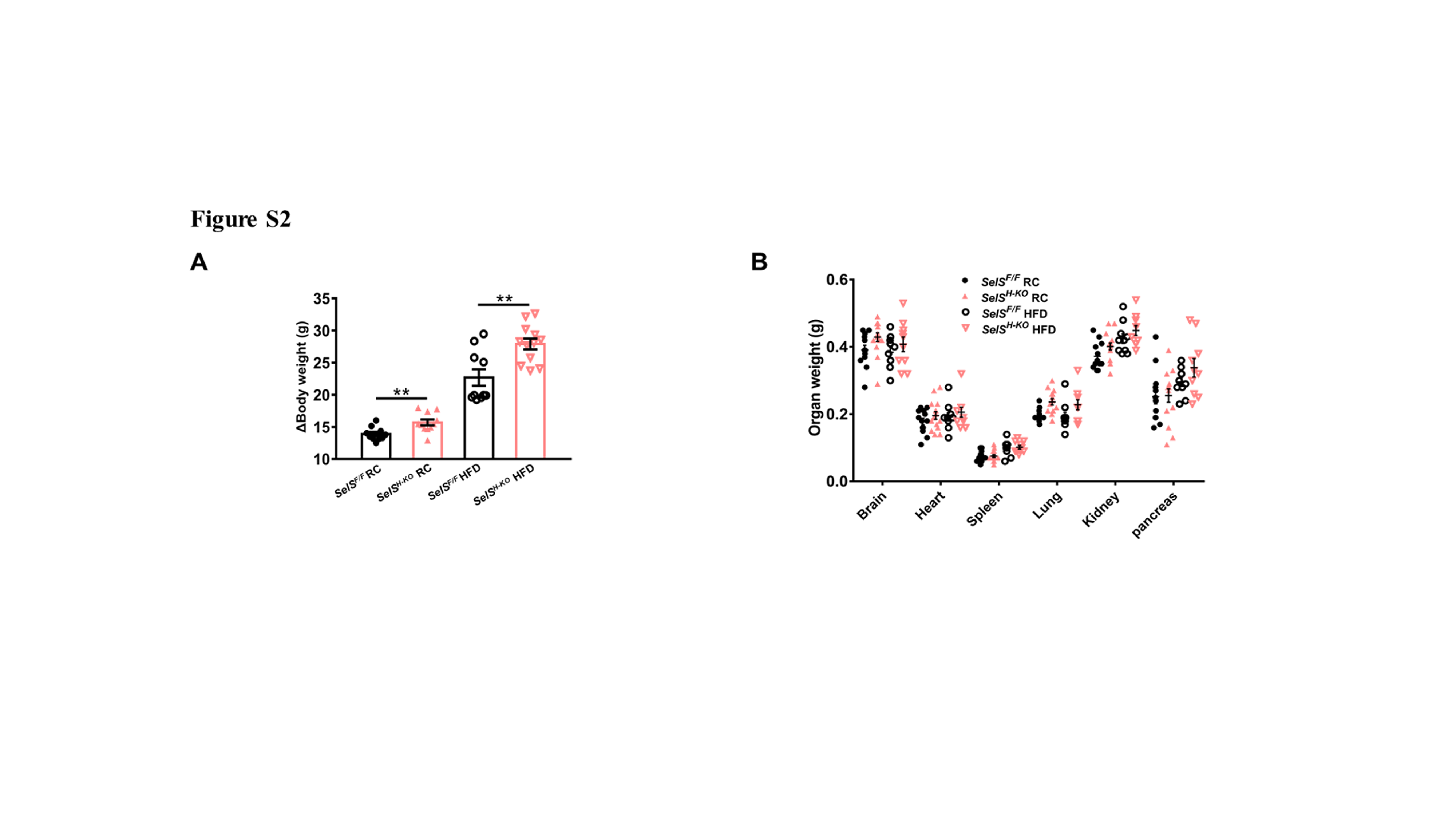

Supplement: Supplementary file 4 — Figure S2 [file 41419_2022_4716_MOESM4_ESM.tif]

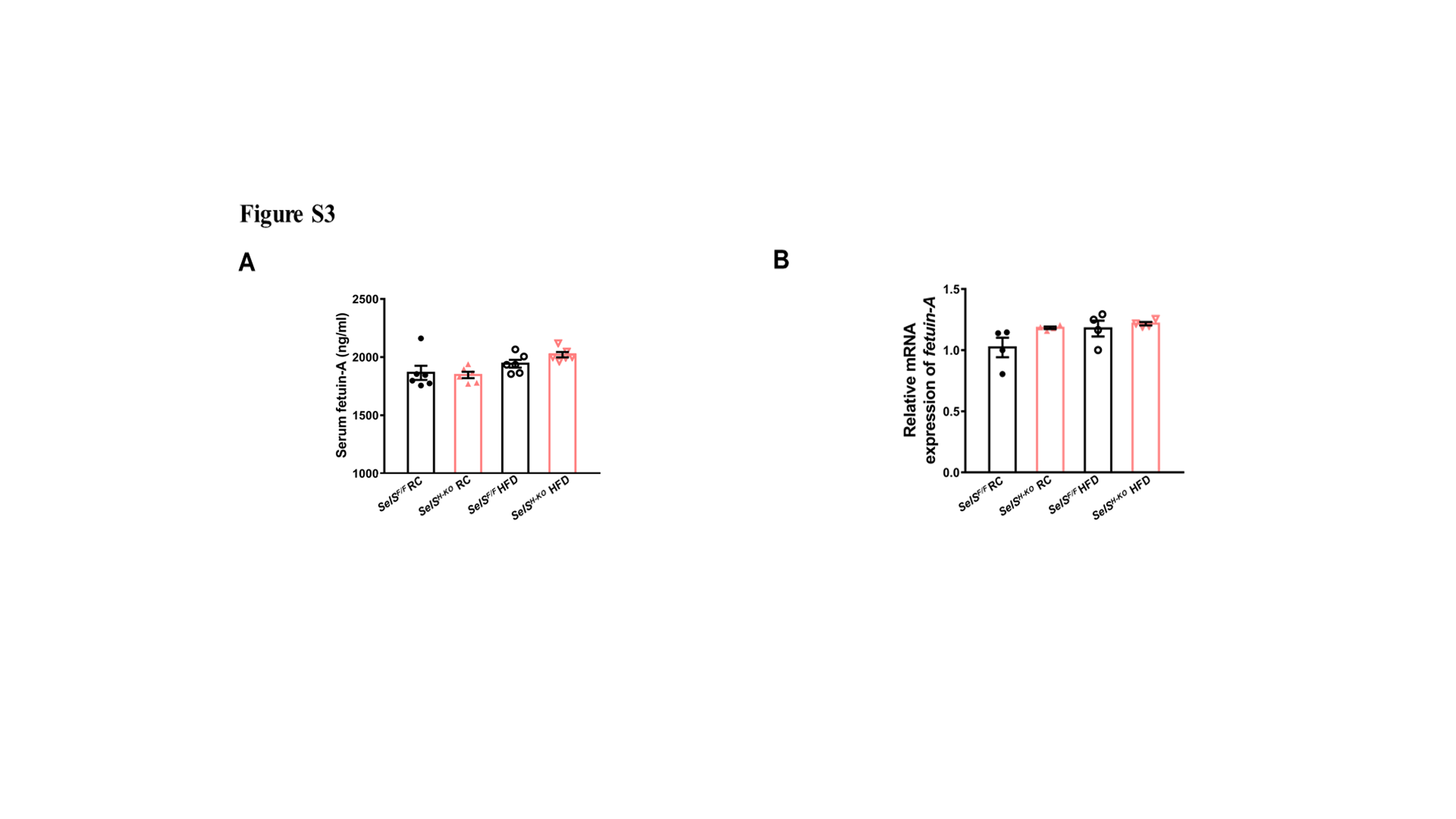

Supplement: Supplementary file 5 — Figure S3 [file 41419_2022_4716_MOESM5_ESM.tif]

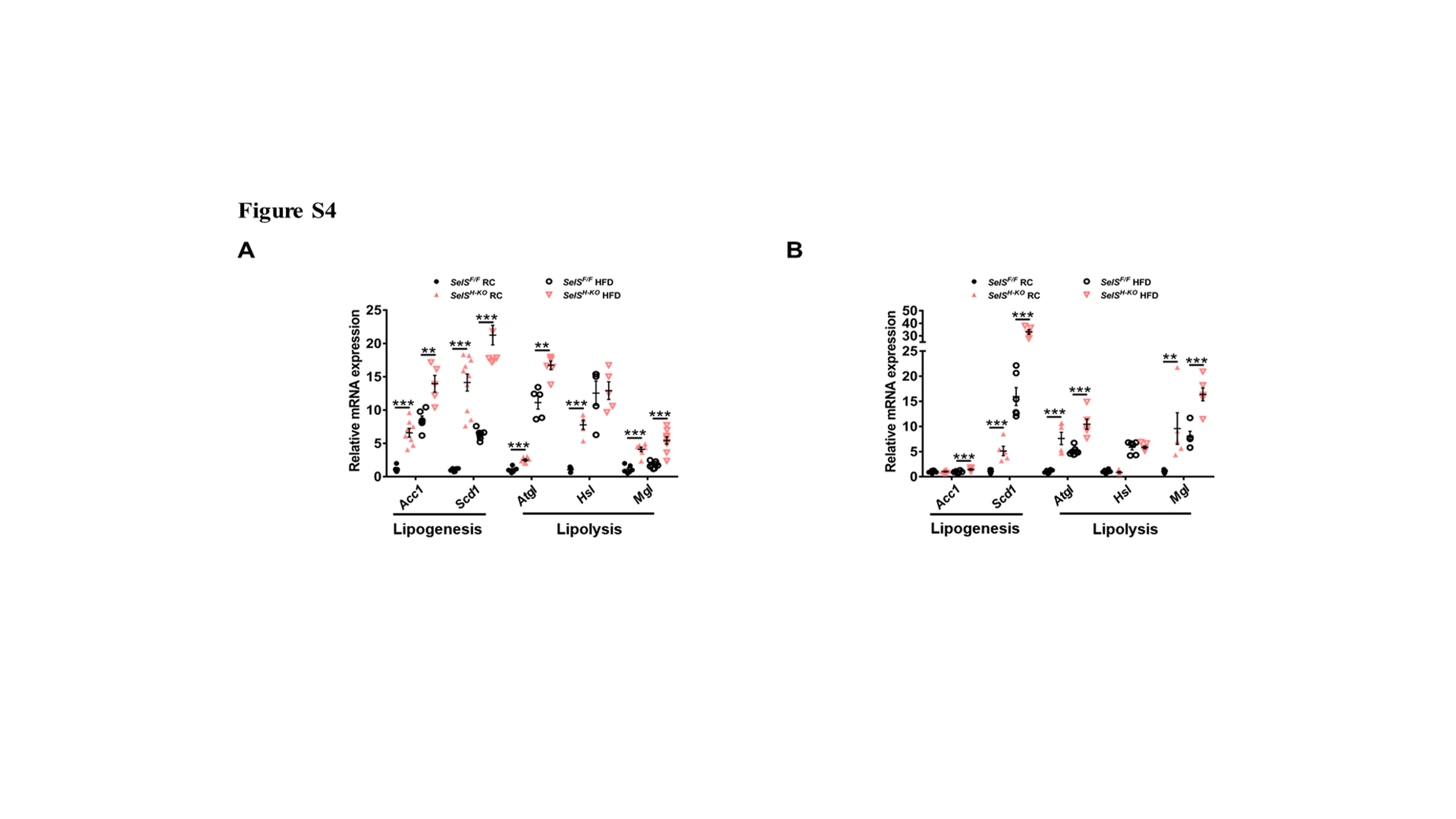

Supplement: Supplementary file 6 — Figure S4 [file 41419_2022_4716_MOESM6_ESM.tif]
