# Supplementary material for: Hepatic deficiency of selenoprotein S exacerbates hepatic steatosis and insulin resistance
Source: Cell Death Dis. 2022 Mar 28;13(3):275. doi: 10.1038/s41419-022-04716-w (PMC8960781; doi:10.1038/s41419-022-04716-w)
Supplement: Supplementary file 7 — Tables [file 41419_2022_4716_MOESM7_ESM.docx]

**Table S1.** Primer sequences for PCR genotyping. Related to Figure S1.

| Gene | *Forward* | *Reverse* |
| --- | --- | --- |
| *Alb-Cre* | *5‘-AATGCTTCTGTCCGTTTGCCGG-3’* | *5‘-CCAGGCTAAGTGCCTTCTCTACA-3’* |
| *SelS* | *5‘-GGGCACACAGTTCTGTTGAGT-3’* | *5'-GCGCTCGTAACCACTGAGAAT-3’* |

|  |
| --- |

**Table S2.** Primary antibodies for Western blotting. Related to Figure 1, 2, 3, 4, 5, 6, 7 and S1.

| Antibody | Cat No. | Manufacturer |
| --- | --- | --- |
| Tubulin | ABP0128 | Abbkine |
| GAPDH | A01021 | Abbkine |
| SelS | HPA010025 | Sigma |
| PCK1 | 16754-1-AP | Proteintech |
| G6Pase | 22169-1-AP | Proteintech |
| GYS2 | 22371-1-AP | Proteintech |
| GCK | [19666-1-AP](http://www.ptgcn.com/products/GCK-Antibody-19666-1-AP.htm) | Proteintech |
| GLUT2 | 20436-1-AP | Proteintech |
| CD36 | 18836-1-AP | Proteintech |
| FATP2 | 14048-1-AP | Proteintech |
| FATP5 | bs-4088R | Bioss |
| FABP1 | 13626-1-AP | Proteintech |
| PPARα | 15540-1-AP | Proteintech |
| SREBP1 | 14088-1-AP | Proteintech |
| ACC1 | ABP54740 | Abbkine |
| ATGL | 55190-1-AP | Proteintech |
| PKCɛ | Ab124806 | Abcam |
| Na^+^/K^+^-ATPase | 14418-1-AP | Proteintech |
| p-IRS1^Tyr896^ | ABP54927 | Abbkine |
| IRS1 | ABP54930 | Abbkine |
| p-Akt^Ser473^ | 4060 | Cell Signaling Technology |
| Akt | ABP0059 | Abbkine |
| p-FOXO1^Ser256^ | 9461 | Cell Signaling Technology |
| FoxO1 | ABP0152 | Abbkine |
| p-GSK3β^Ser9^ | ABP0037 | Abbkine |
| GSK3β | ABP51488 | Abbkine |
| p-IRE1α^Ser724^ | PA1-16927 | Invitrogen |
| IRE1α | PA5-20189 | Invitrogen |
| GRP78 | 3177 | Cell Signaling Technology |
| XBP1s | 24868-1-AP | Proteintech |
| p-eIF2α^Ser51^ | Ab32157 | Abcam |
| eIF2α | 11170-1-AP | Proteintech |
| CHOP | 15204-1-AP | Proteintech |
| p-JNK^Thr183+Tyr185^ | Ab131499 | Abcam |
| JNK | 24164-1-AP | Proteintech |
| Adiponectin | 21613-1-AP | Proteintech |

**Table S3.** Primer sequences for real-time quantitative PCR. Related to Figure 1, 3, 4, 5, 7, S1, S3 and S4.

| Gene | *Forward* | *Reverse* |  |
| --- | --- | --- | --- |
| *SelS* | *5'-AGCCGAGACTGTTCTGGA-3’* | *5'-GGCATTTAGATCTTCCTGCATT-3’* |  |
| *Pck1* | *5'-AGCATTCAACGCCAGGTTC-3’* | *5'- CGAGTCTGTCAGTTCAATACCAA-3’* |  |
| *G6pc* | *5'-CGACTCGCTATCTCCAAGTGA-3’* | *5'-GGGCGTTGTCCAAACAGAAT-3’* |  |
| *Glut2* | *5'-ACCGGGATGATTGGCATGTT-3'* | *5'-GGACCTGGCCCAATCTCAAA-3'* |  |
| *Gck* | *5'-AGGAGGCCAGTGTAAAGATGT-3'* | *5'-CTCCCAGGTCTAAGGAGAGAAA-3'* |  |
| *Gys2* | *5'-GAGTGGGGAGAGAATTACTTCCT-3'* | *5'-GGGCTCACATTGTTCTACTTGA-3'* |  |
| *Cd36* | *5'- TTGAAAAGTCTCGGACATTGAG-3’* | *5'-TCAGATCCGAACACAGCGTA-3’* |  |
| *Fatp2* | *5'- GATGCCGTGTCCGTCTTTTAC-3’* | *5'-GACTTCAGACCTCCACGACTC-3’* |  |
| *Fatp5* | *5'-GTTCTCCCGTCCAAGACCATT-3’* | *5'-GCTCCGTACAGAGTGTAGCAAG-3’* |  |
| *Fabp1* | *5'-GTCAGAAATCGTGCATGAAGGG-3’* | *5'-GAACTCATTGCGGACCACTTT-3’* |  |
| *Pparα* | *5'-AGAGCCCCATCTGTCCTCTC-3'* | *5'- ACTGGTAGTCTGCAAAACCAAA-3'* |  |
| *Cpt2* | *5'-CAAAAGACTCATCCGCTTTGTTC-3’* | *5'-CATCACGACTGGGTTTGGGTA-3’* |  |
| *Acox1* | *5'-TAACTTCCTCACTCGAAGCCA-3’* | *5'-AGTTCCATGACCCATCTCTGTC-3’* |  |
| *Srebp1c* | *5'- TGCGGCTGTTGTCTACCATA-3’* | *5'-TGCTGGAGCTGACAGAGAAA-3’* |  |
| *Acc1* | *5'-GGAGAAACCTGCCAAGTATG-3'* | *5'-CCTGCCTGTCTCCATCCA-3'* |  |
| *Scd1* | *5'-TGGAAATGCCTTTGAGATGG-3'* | *5'-CCAGCCAGCCTCTTGACTAT-3'* |  |
| *Atgl* | *5'-GCTGTGGAATGAGGACATAGGA-3'* | *5'-GCATAGTGAGTGGCTGGTGAA-3'* |  |
| *Hsl* | *5'-TGTGTCAGTGCCTATTCAG-3'* | *5'-GAACAGCGAAGTGTCTCT-3'* |  |
| *Mgl* | *5'-GCTGTGGAATGAGGACATAGGA-3'* | *5'-GCATAGTGAGTGGCTGGTGAA-3'* |  |
| *Fgf21* | *5'-CTGCAGCTGAAAGCCTTGAAGC-3'* | *5'-GTATCCGTCCTCAAGAAGCAGC-3'* |  |
| *Fetuin-A* | *5'-CCACATCCTGAAACAAGACGG-3'* | *5'-GCACAACTTACGAACGTCCTCT-3'* |  |
| *Adipon-ectin* | *5'-CAACCAACAGAATCATTATG-3'* | *5'-GGTAAGAGAAGTAGTAGAGT-3'* |  |
| *Pparγ* | *5'-TGTGGACCTCTCCGTGATGG-3'* | *5'-GGTTCTACTTTGATCGCACTTTGG-3'* | |
| *C/ebpα* | *5'-GCGGGAACGCAACAACATC-3'* | *5'-GTCACTGGTCAACTCCAGCAC-3'* | |
| *Gapdh* | *5'-GGGCTGGCATTGCTCTCAATG-3'* | *5'-CATGTAGGCCATGAGGTCCAC-3'* | |
