# Supplementary material for: Hepatic deficiency of selenoprotein S exacerbates hepatic steatosis and insulin resistance
Source: Cell Death Dis. 2022 Mar 28;13(3):275. doi: 10.1038/s41419-022-04716-w (PMC8960781; doi:10.1038/s41419-022-04716-w)

**Figure 1**

**Figure 1B**

**SelS (the middle 6 panels)**

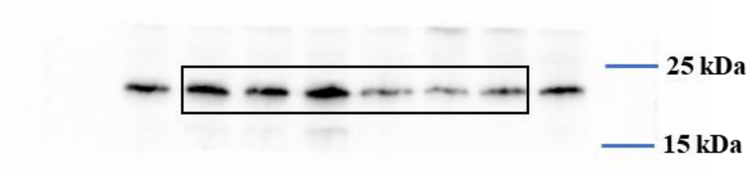


**Tubulin (the middle 6 panels)**

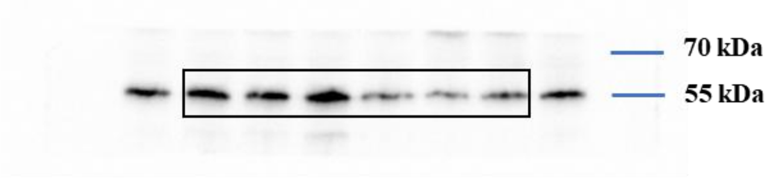


**Figure 1C**

**SelS**

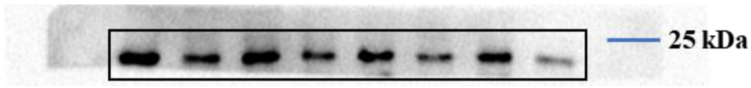


**GAPDH**

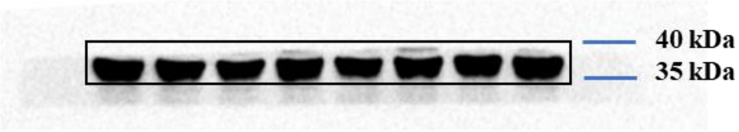


**Figure 1D**

**SelS**

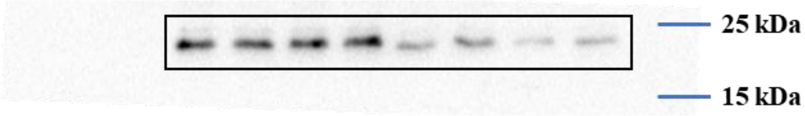


**Tubulin**

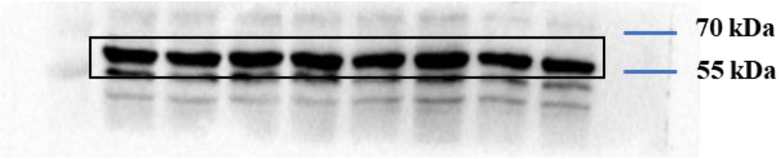

**Figure 2**
**Figure 2B**
**SelS**

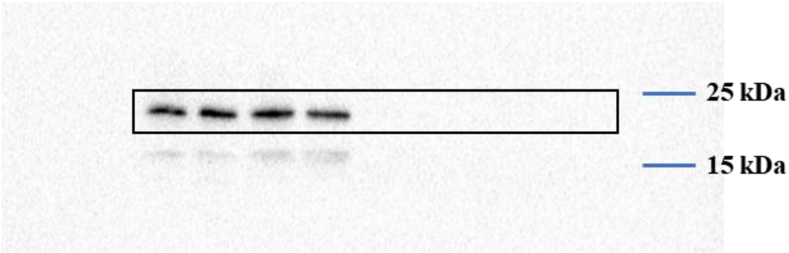


**Tubulin**

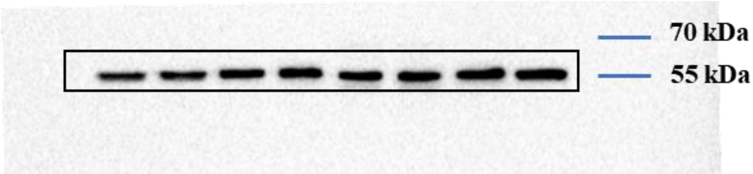

**Figure 3**

**Figure 3D**

**CD36**

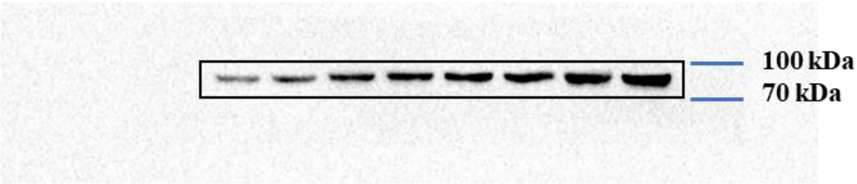


**FATP2**

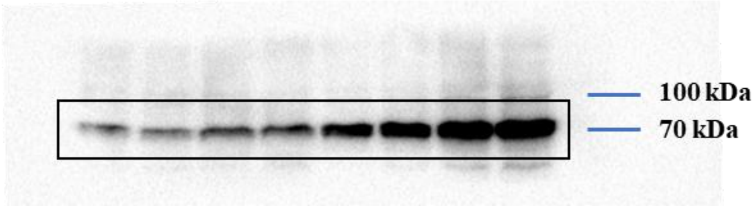


**FATP5**

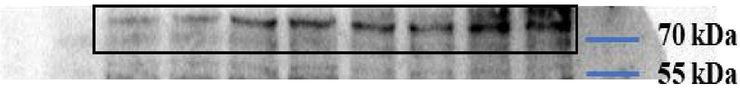


**FABP1**

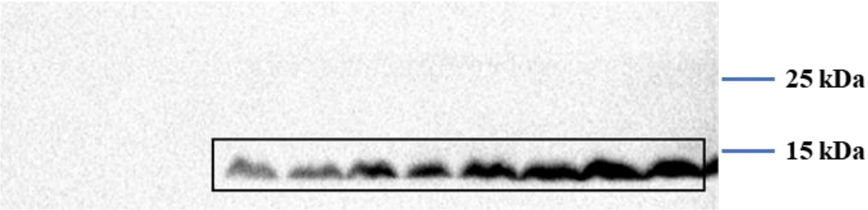


**PPARα**

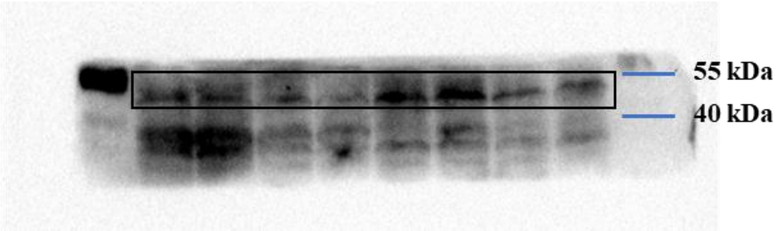


**SREBP1**

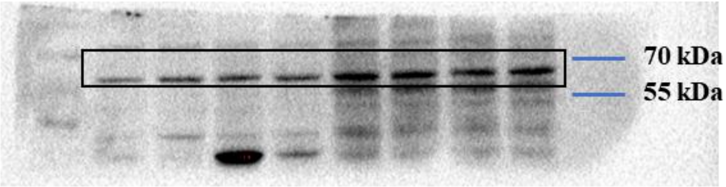

**ACC1**

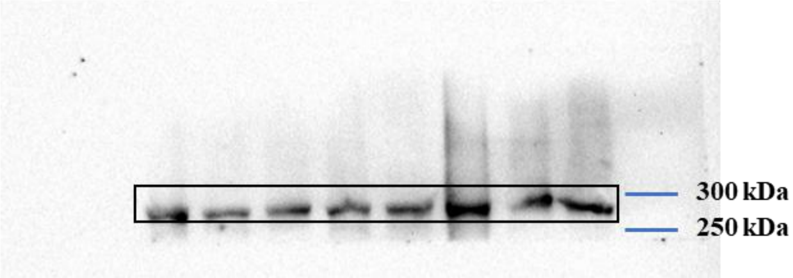


**ATGL**

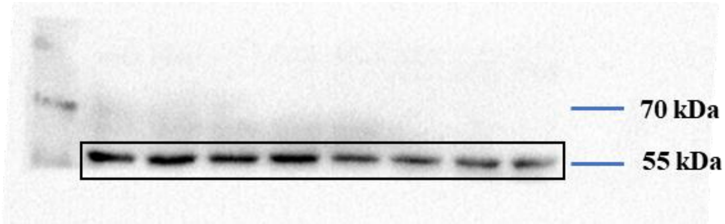


**Tubulin（2 bands）**

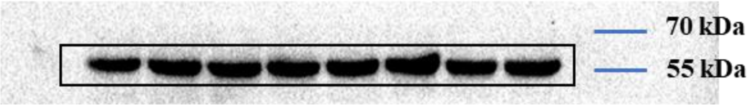


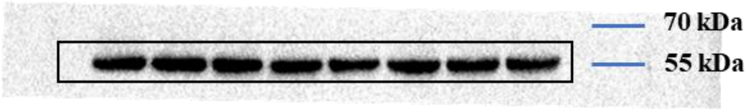


**Figure 3E**
**GRP78**

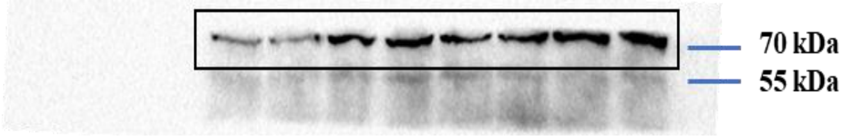


**p-IRE1α**

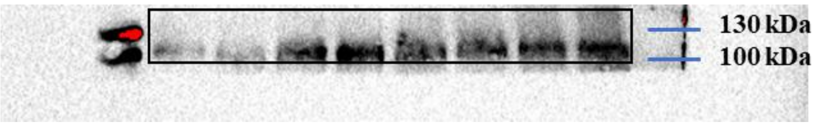


**IRE1α**

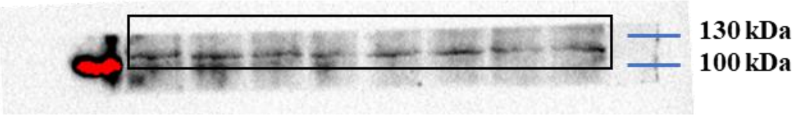

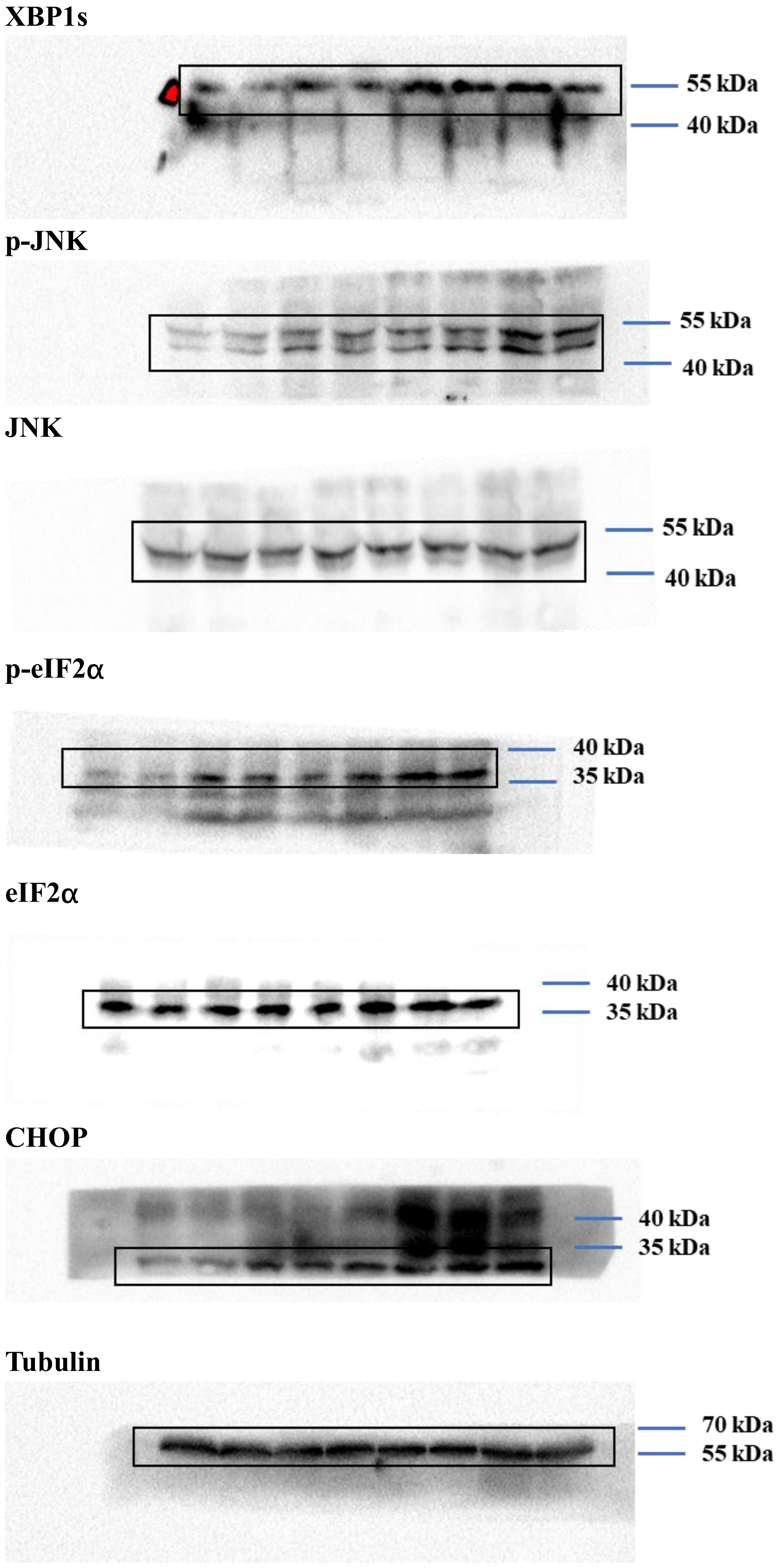
XBP1s
55 kDa
40 kDa
p-JNK
55 kDa
40 kDa
JNK
55 kDa
40 kDa
p-eIF2α
40 kDa
35 kDa
eIF2α
40 kDa
35 kDa
CHOP
40 kDa
35 kDa
Tubulin
70 kDa
55 kDa

**Figure 4**
**Figure 4G**
**PCK1**

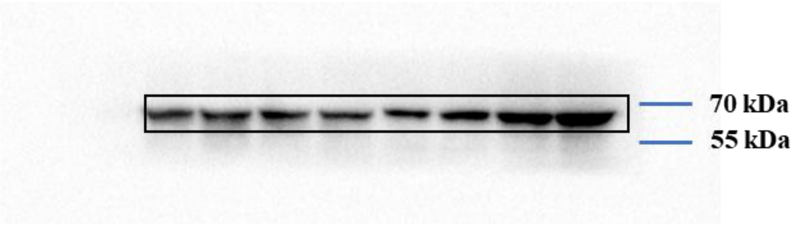


**G6Pase**

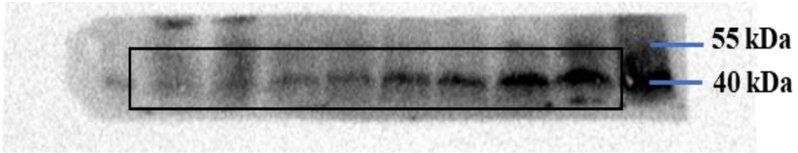


**GYS2**

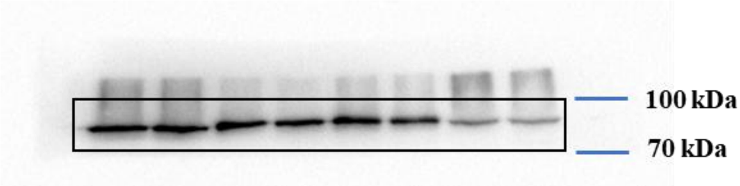


**GCK**

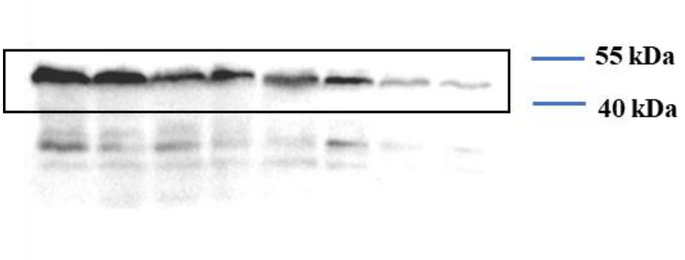


**GLUT2**

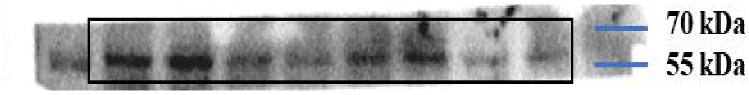


**Tubulin**

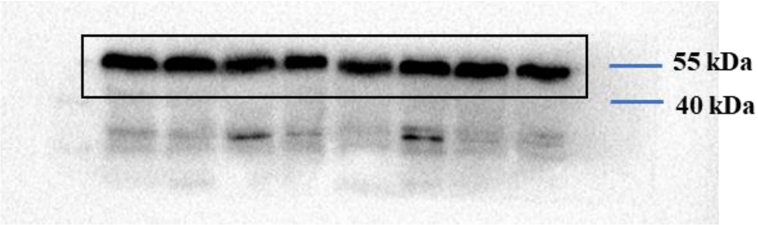

**Figure 4H**

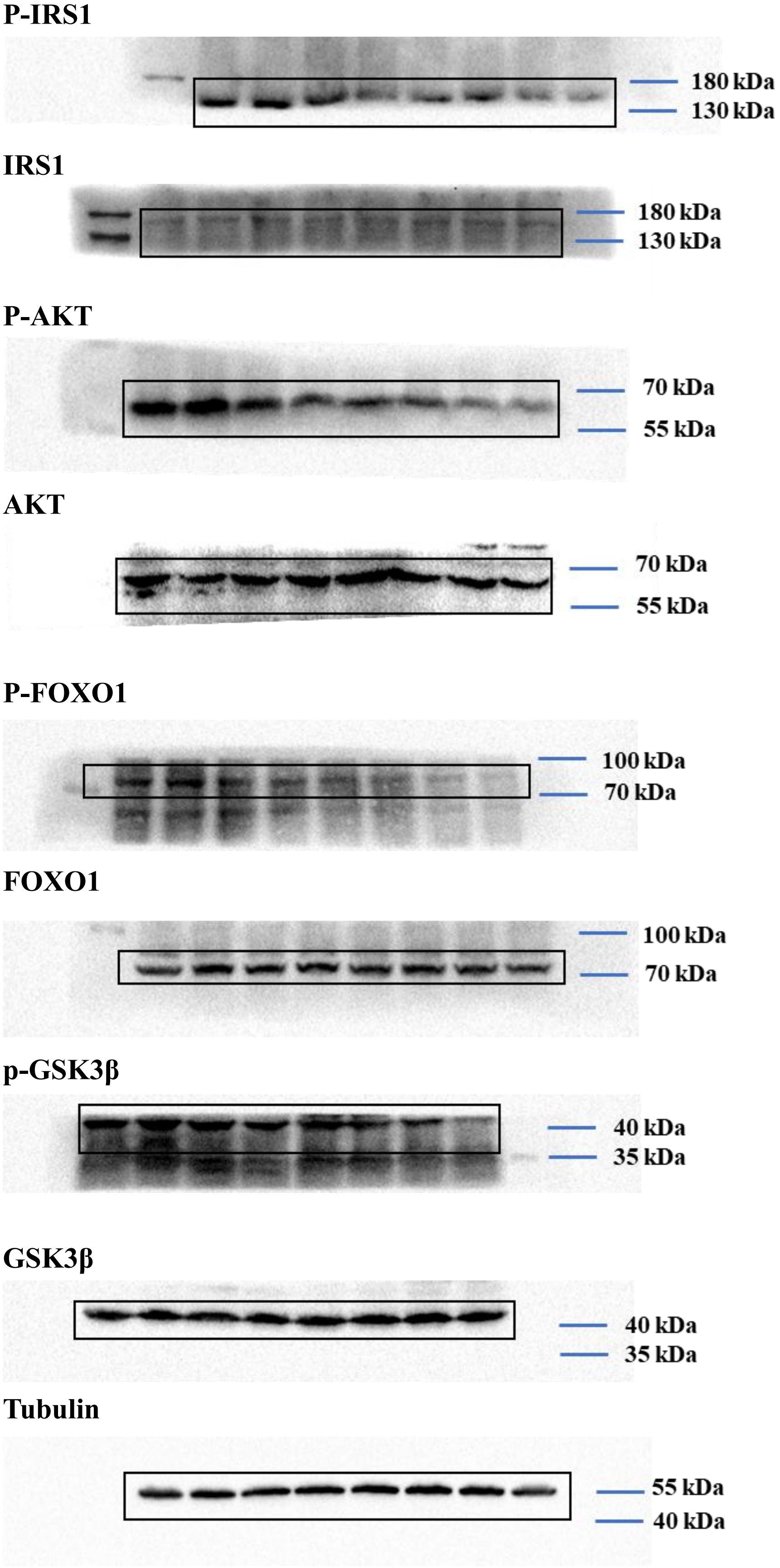
P-IRS1
180 kDa
130 kDa
IRS1
180 kDa
130 kDa
P-AKT
70 kDa
55 kDa
AKT
70 kDa
55 kDa
P-FOXO1
100 kDa
70 kDa
FOXO1
100 kDa
70 kDa
p-GSK3β
40 kDa
35 kDa
GSK3β
40 kDa
35 kDa
Tubulin
55 kDa
40 kDa

**Figure 4I**

**Membrane PKCε**

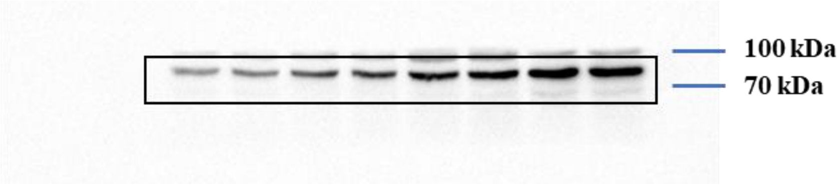


**Na + K + -ATPase**

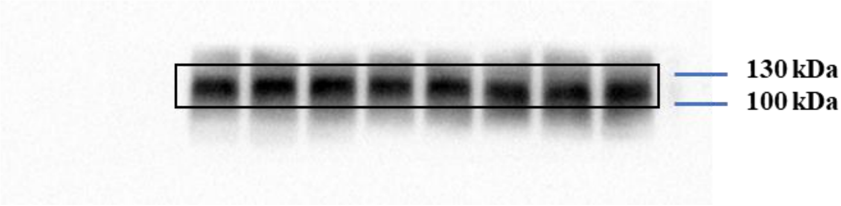


**Cytosol PKCε**

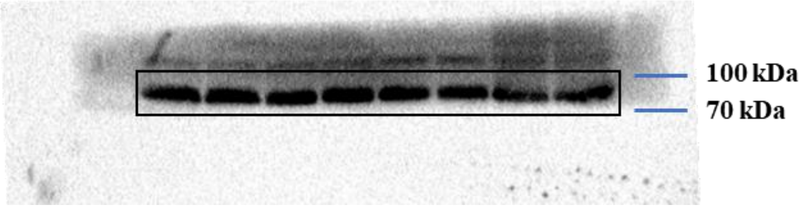


**Tubulin**

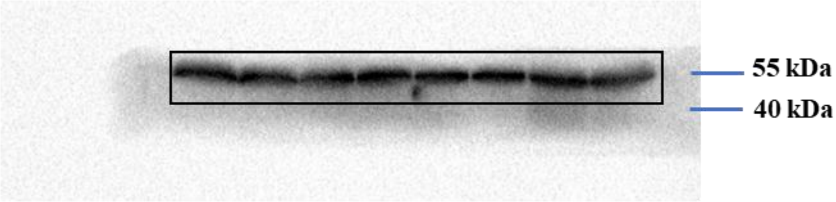

Figure 5

Figure 5A

KD-SelS

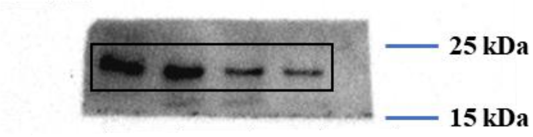


KD-Tubulin

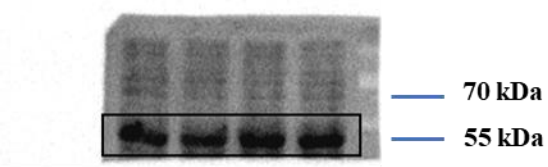


OE-SelS (the left three panels)

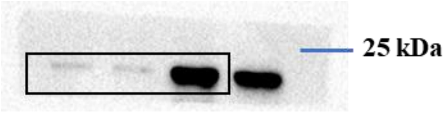


OE-Tubulin(the left three panels)

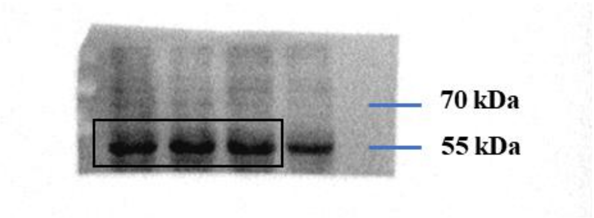


Figure 5F

GRP78

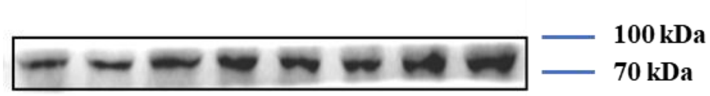


p-IRE1α

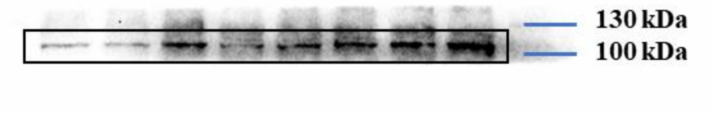


IRE1α

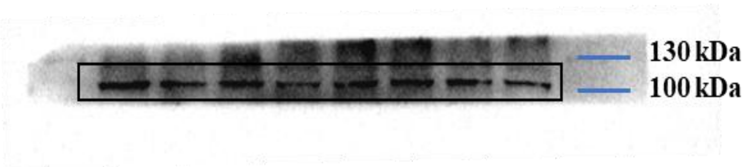

**p-JNK**

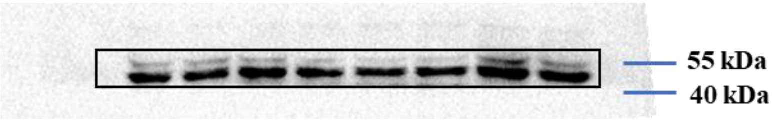


**JNK**

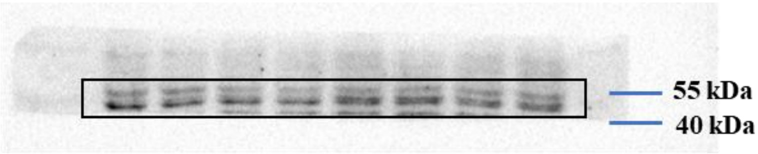


**p-eIF2α**

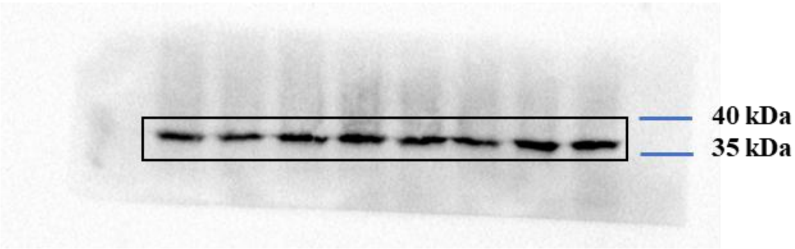


**eIF2α**

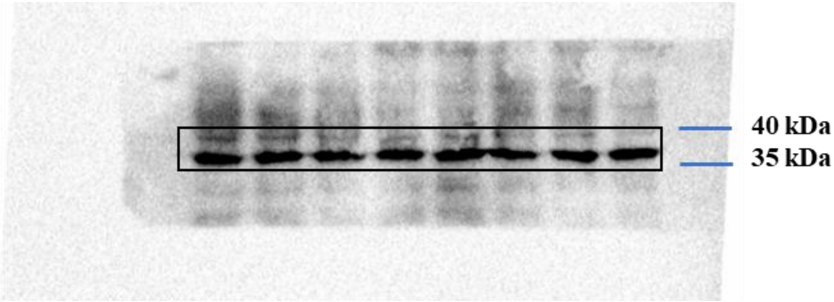


**CHOP**

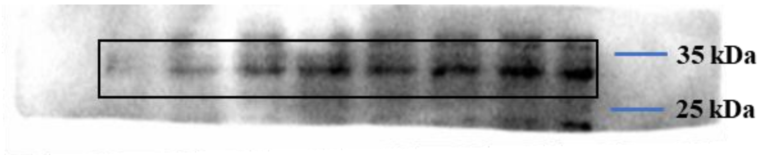


**Tubulin**

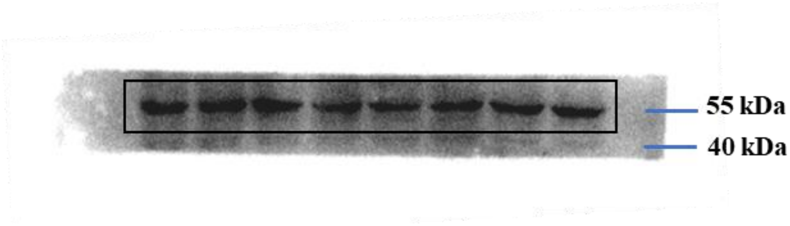

**Figure 5G**

**GRP78**

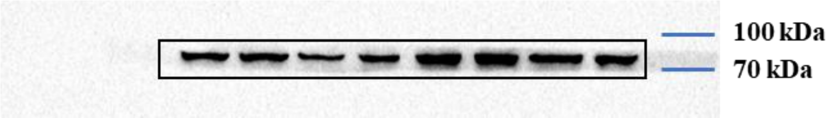


**p-IRE1α**

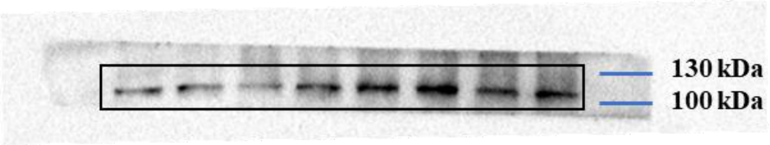


**IRE1α**

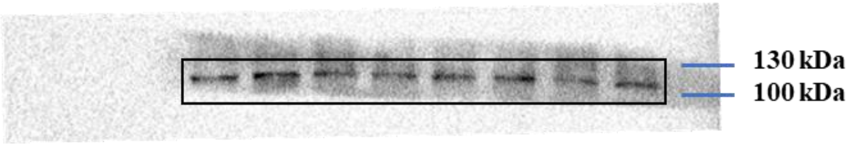


**p-JNK**

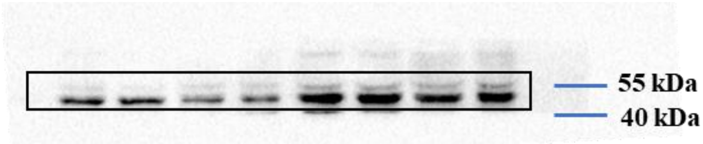


**JNK**

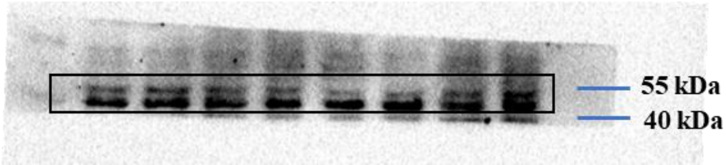


**p-eIF2α**

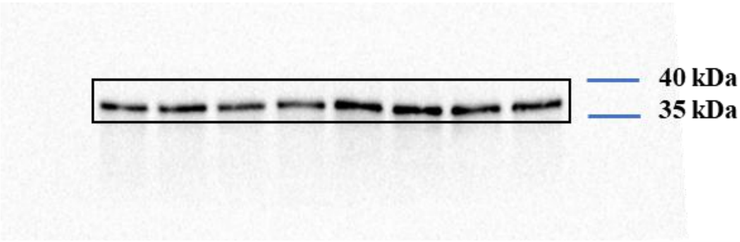


**eIF2α**

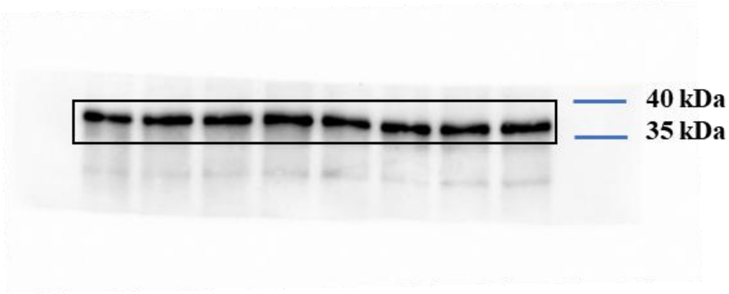

**CHOP**

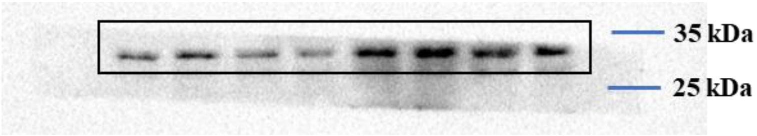


**Tubulin**

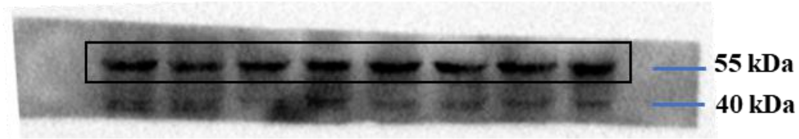

**Figure 6**

**Figure 6A**

**p-IRS1**

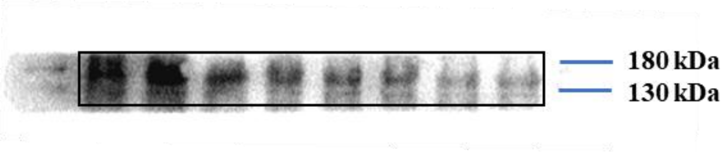


**IRS1**

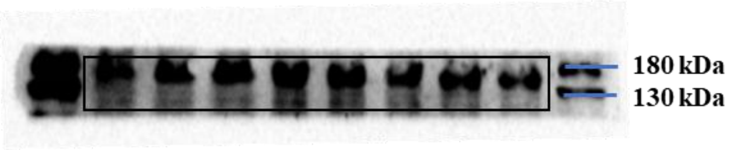


**p-Akt**

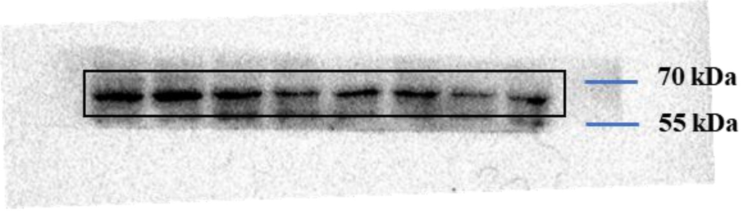


**Akt**

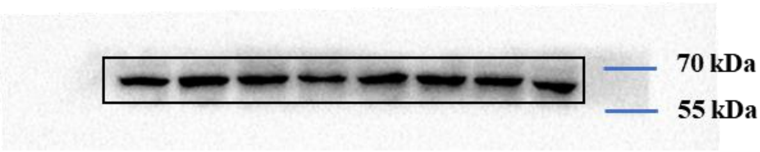


**GAPDH**

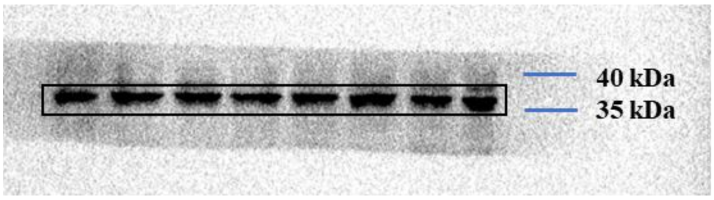


**Figure 6B**

**p-IRS1**

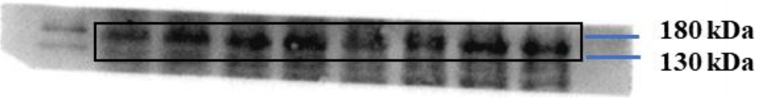


**IRS1**

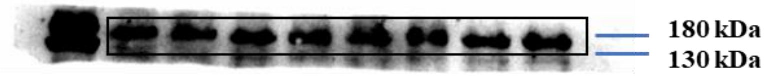


**p-Akt**

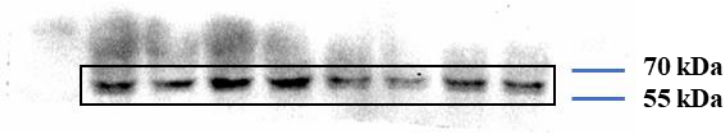

**Akt**

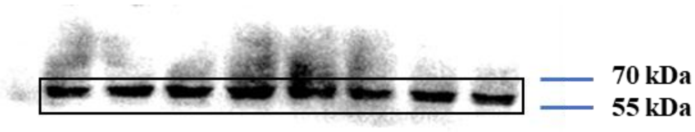


**GAPDH**

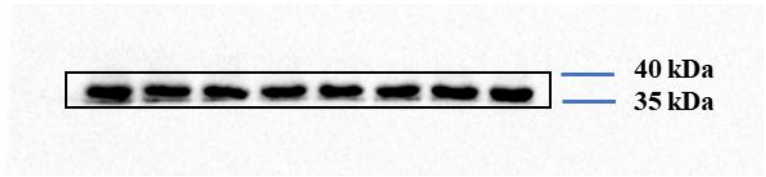


**Figure 6C**

**Membrane PKCε**

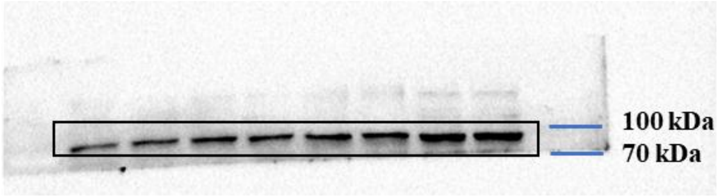


**Na + K + -ATPase**

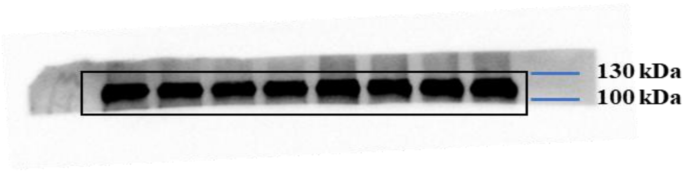


**Cytosol PKCε**

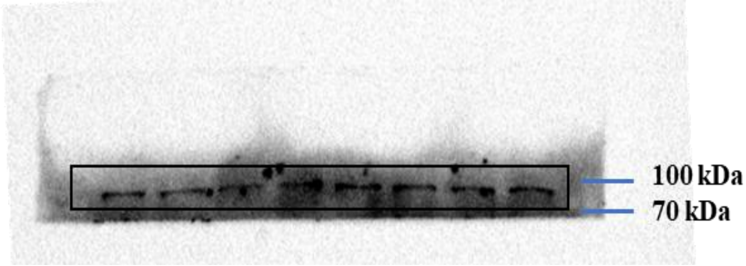


**GAPDH**

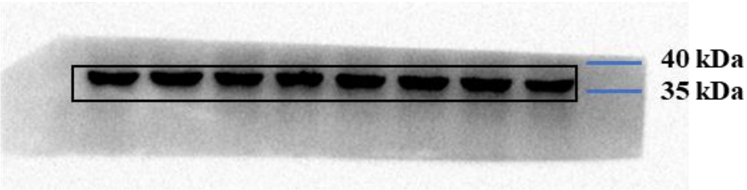

**Figure 6D**
**Membrane PKCε**

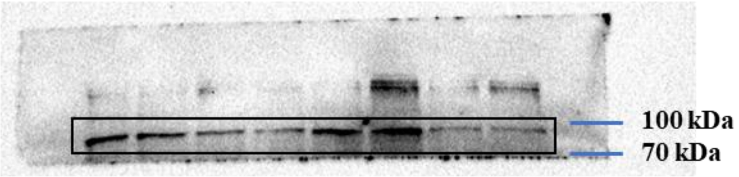


**Na + K + -ATPase**

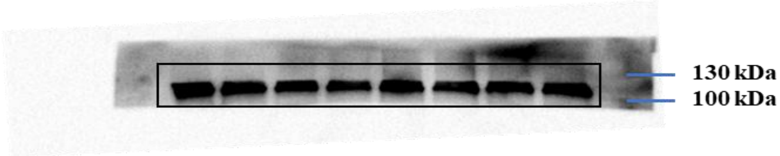


**Cytosol PKCε**

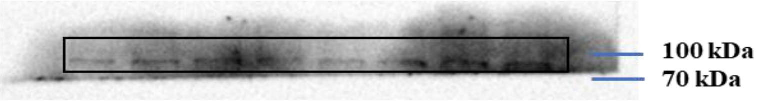


**GAPDH**

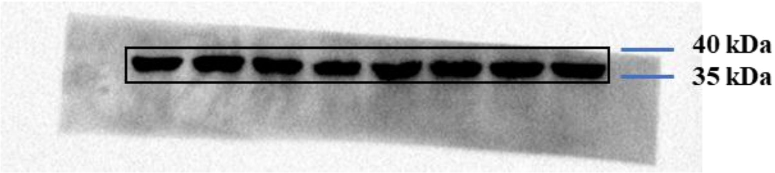


**Figure 6E**
**p-IRS1**

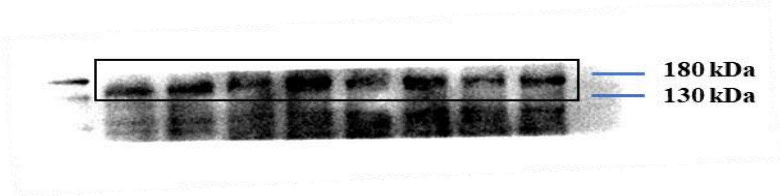


**IRS1**

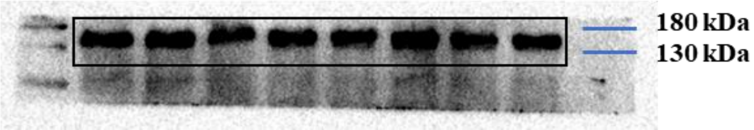


**GAPDH**

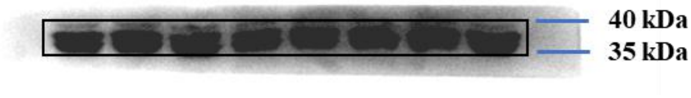

Figure 6F

p-Akt

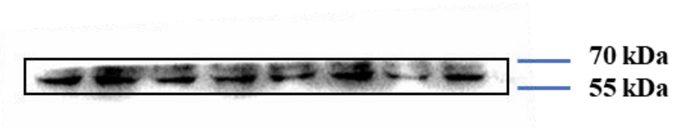


Akt

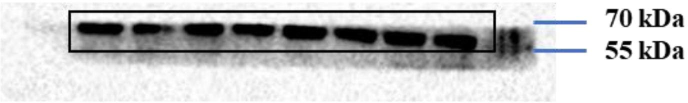


GAPDH

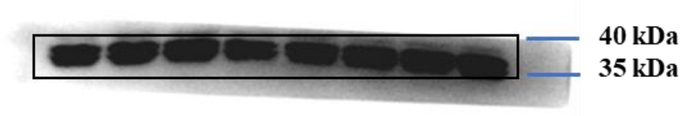


Figure 6G

p-IRS1

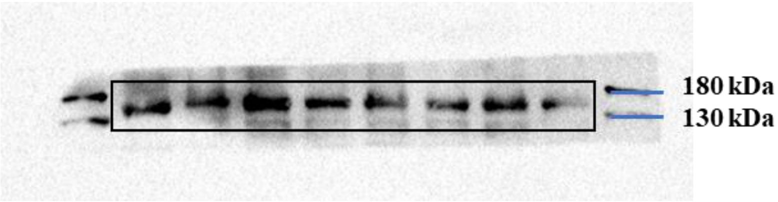


IRS1

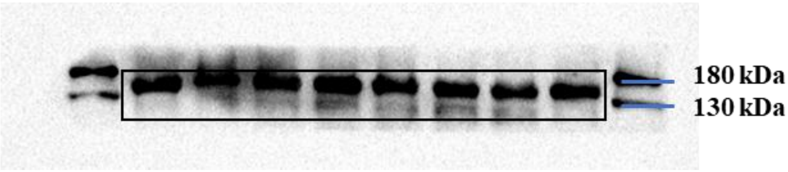


GAPDH

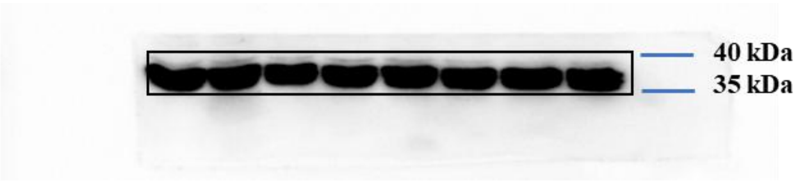


Figure 6H

p-Akt

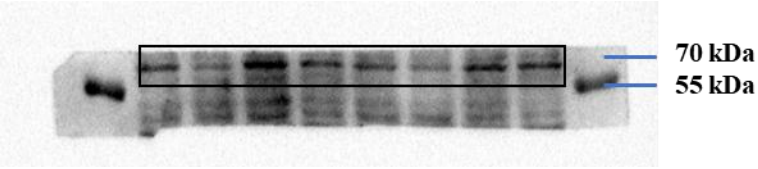

**Akt**

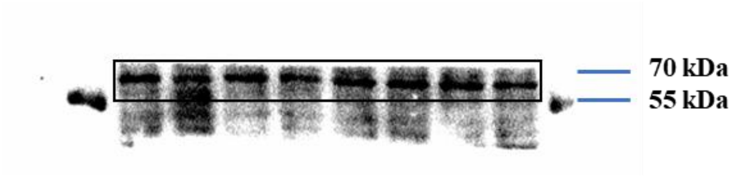


**GAPDH**

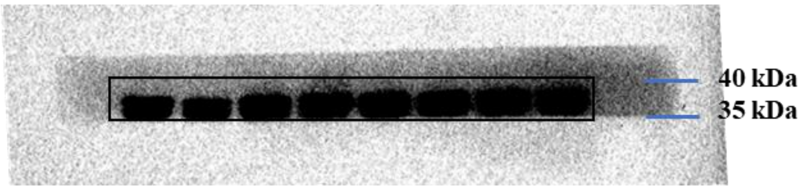

**Figure 7**

**Figure 7I**

**SelS**

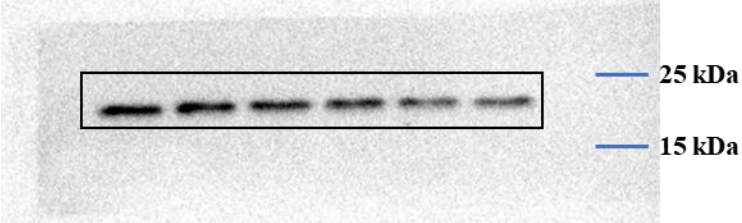


**Adiponectin**

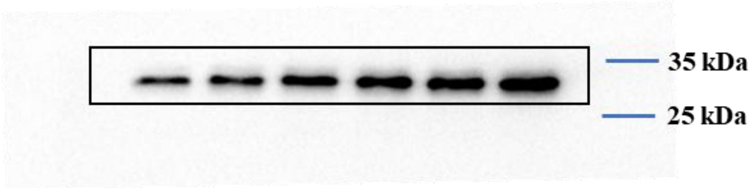


**Tubulin**

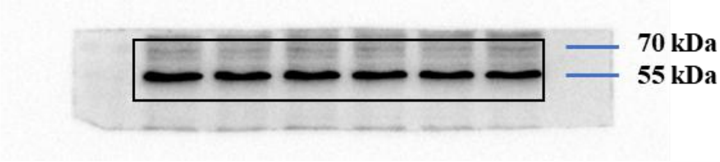


**Figure 7J**

**SelS**

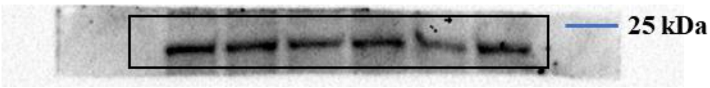


**Adiponectin**

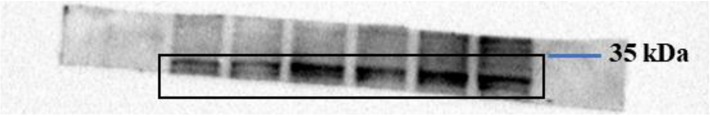


**Tubulin**

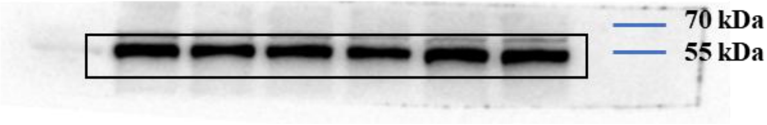

**Supplemental Figure 1D**

**SelS**

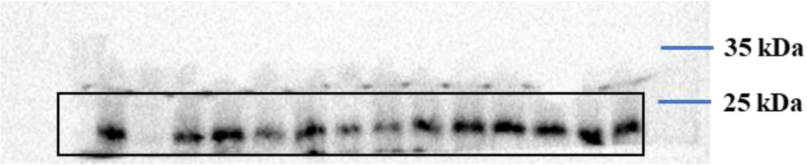


**Tubulin**

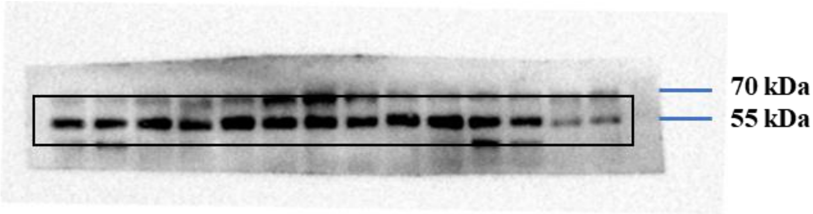

Supplement: Supplementary file 8 — Uncut Western blots [file 41419_2022_4716_MOESM8_ESM.pdf]
